# How Thermodynamic, Electronic, and Steric Factors Influence Mesitylcopper Oligomers

**DOI:** 10.1021/acs.jpca.5c04666

**Published:** 2025-10-24

**Authors:** D.P. Ngan Le, Michael Stollenz, Samer Gozem

**Affiliations:** † Department of Chemistry, 1373 Georgia State University, Atlanta, Georgia 30303, United States; § Department of Chemistry and Biochemistry, 15617 Kennesaw State University, Kennesaw, Georgia 30144, United States

## Abstract

Mesitylcopper (CuMes) is a highly versatile organocopper reagent used in both organic and inorganic syntheses. It has previously been shown that CuMes exists as a tetrameric or pentameric cyclic oligomer [CuMes]*_n_* (*n* = 4, 5), both in solution and in the solid state. The bonding arrangement between the [CuMes] units has qualitatively been described as localized three-center two-electron (3c-2e) bonds. However, the electronic, structural, and thermodynamic forces driving this aggregation are still not well understood. For this reason, we employed density functional theory (DFT) calculations to study mesitylcopper as a monomeric [CuMes] unit and [CuMes]*_n_* oligomers with *n* = 2 to *n* = 7. We found that there is a strong electronic driving force for aggregation caused by strong mixing between the Cu’s *d* orbitals and Mes’s π orbitals in oligomers larger than the dimer. This mixing is only optimized in oligomers with *n* ≥ 3, where the mesityl group is no longer bonded to a single copper center but instead becomes a bridging ligand. Beyond the trimer, steric and entropic factors become relevant for determining the relative stabilities of the different aggregates, with midsized oligomers (*n* = 4–5) having the optimal balance between the electronic Cu–C bonding character, Cu···Cu attractive forces, entropy, reduced internal ring strain, and reduced steric interactions between the mesityl groups.

## Graphical abstract

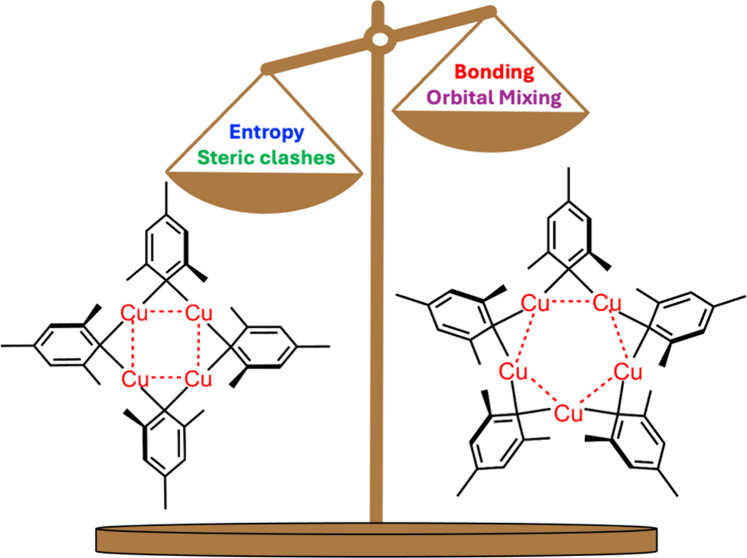

## Introduction

Mesitylcopper Cu­(I) (CuMes, Scheme 1), one of the few well-defined homoleptic arylcopper compounds, has a broad application range in organic cuprate catalysis, as a synthon for photoluminescent clusters and biorelevant copper­(I) complexes, and as an efficient precursor for nanoparticles and intermetallic phases.^1^

**Scheme 1:**
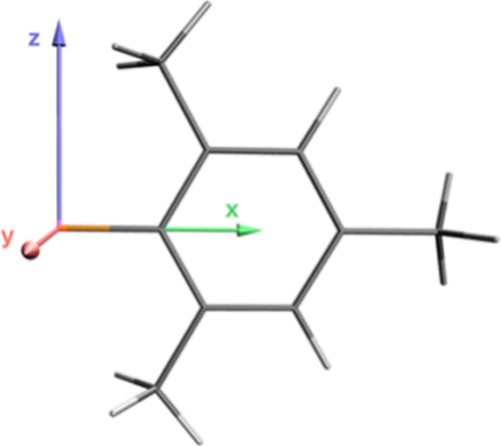
Monomer Structure and x, y, z Axes Used for Constructing the Oligomers

Organocopper chemistry has been dominated by ionic cuprates and their applications in catalytic or stoichiometric C–C and C–heteroatom bond forming transformations such as substitutions, conjugate additions, cross-coupling reactions, and carbocuprations.^2–4^ On the other hand, the synthetic utility of neutral organocopper compounds (RCu) is typically more limited. This is because of their thermal instability and low solubility. Alkylcopper compounds in particular are thermodynamically less stable than their aryl counterparts; for instance, methylcopper (MeCu)^5–7^ starts to decompose at temperatures above ∼ – 25 °C while phenylcopper (CuPh)^8,9^ remains stable up to ∼ 100 °C. However, CuPh suffers from kinetic lability and is insoluble in common organic solvents. Even though CuPh was reported for the first time more than 100 years ago, its molecular structure is still unknown but likely to be of polymeric nature.^8,9^ Introducing sterically demanding substituents in the *o*-phenyl positions results in a significant kinetic stabilization, which leads to isolable oligomers [CuAr]*_n_* but no mononuclear arylcopper species (e.g., see Figure 1).^10–14^ The degree of aggregation depends on the steric bulk of these substituents and allows for the formation of dimers (*n* = 2) in case of sterically demanding terphenyl groups (**III**).^15^ Among these well-characterized arylcopper compounds, CuMes (**I** and **II**) has become most popular for synthetic applications because of its high kinetic stabilization and increased solubility through three methyl substituents, which also serve as useful sensors in ^1^H and ^13^C NMR spectroscopy. We have utilized CuMes as a clean Cu^I^ source for a series of photoluminescent bis­(amidinate) clusters,^16–18^ which can also retain a reactive mesitylcopper site.^19^

**Figure 1:**
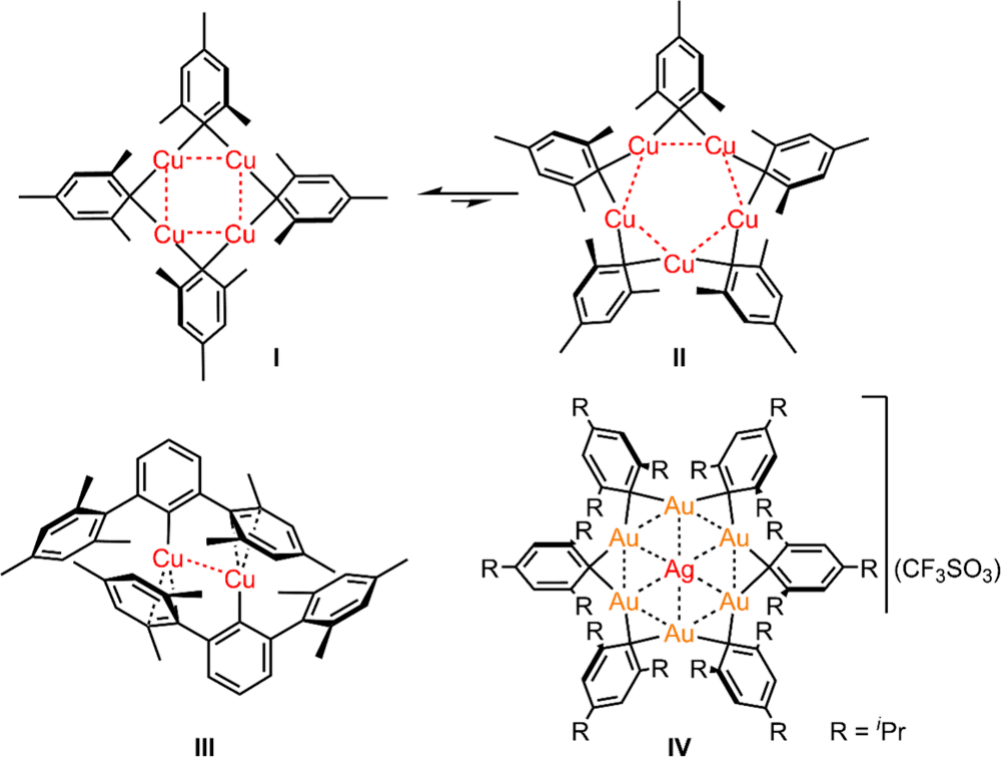
Examples of aryl-coinage metal oligomeric clusters.

CuMes has been observed in solution and in the solid state only as cyclic oligomers or as monomers being stabilized by additional ligands.^1^ Single-crystal X-ray diffraction and ^1^H NMR measurements have shown that CuMes forms tetramers [CuMes]_4_ (**I**) and pentamers [CuMes]_5_ (**II**) in solution.^1,10–12^ Their crystal structures feature a cyclic arrangement of the copper atoms with the mesityl groups acting as μ-bridging ligands, each connecting two adjacent copper atoms in a (3c-2e) bond. As more recently demonstrated by a series of heteroleptic mesitylcopper/PNNP pincer ligand heteroleptic complexes, this symmetric 3c-2e bond can be converted into an unsymmetric 2c-2e bond that is more similar to aryl-cuprate type structures.^20^ Due to the short Cu^I^···Cu^I^ distances (∼ 2.42 Å on average in [CuMes]_4_),^12^ significant d^10^···d^10^ contact interactions exist in mesitylcopper that support the two cyclic structures. No ring sizes of homoleptic [CuMes]*_n_* other than *n* = 4 and 5 have been observed so far. However, in case of Au^I^, the combination of more bulky 2,4,6-triisopropylphenylgold­(I) fragments, centered by a silver­(I) cation, originating from [Ag­(CF_3_SO_3_)], affords a unique hexameric ring in the ionic complex **IV** (Figure 1).^21^

These small CuMes oligomers can be considered as simple model systems for understanding the driving force for Cu^I^ dissociation and Cu–C bond formation in small copper nanoparticles (CuNPs).^22–24^ Furthermore, understanding the thermodynamics behind mesitylcopper aggregation can also prove to be very useful for controlling its reactivity in synthetic applications. Belanzoni et al. have carried out computational studies on cyclic constructs of σ-aryl-bound coinage metals,^25^ which provided important information about the electronic factors and geometries of these systems. They used highly symmetric (D_nh_) models where the mesityl group was replaced by a smaller phenyl group (i.e., CuPh). Herein, we revisit the [CuMes]*_n_* cyclic oligomers with a focus on the thermodynamic, electronic, and structural factors that govern their oligomeric stability.

## Methods – Computational Details

### Molecular Modeling of the CuMes Oligomers

We started by building an energy minimized model of the CuMes monomer having C_S_ point group symmetry. This monomer unit was used to construct proposed ring structures for the [CuMes]*_n_* cyclic oligomers ranging from the dimer (*n* = 2) to the heptamer (*n* = 7) following these steps (Scheme 1):

1) We aligned the monomer so that all heavy atoms are in the xz plane, and such that the Cu was placed at the origin and the Cu–C bond was aligned along the *x* axis.
2) We shifted the position of the Cu atoms along the *x* axis by 1.2 Å.
3) We shifted the entire monomer unit along the *y*-axis by a distance of 1.2/tan­(θ), where θ is equal to 90° (dimer), 60° (trimer), 45° (tetramer), 36° (pentamer), 30° (hexamer), and 26° (heptamer). This displacement ensures that the Cu–C bond length is 1.97 Å and the Cu···Cu separation is 2.4 Å, both distances being within the range of typical Cu–C bonds and Cu···Cu distances.
4) We rotated the monomer around the *z*-axis using the R_Z_ rotation matrix and angles of 2θ, with θ defined above for each oligomer.

The cyclic oligomer model structures constructed in this way all had C_nh_ symmetry where *n* is the number of units in the oligomer. Geometry optimizations were then carried out using the PBE0 (also known as PBE1PBE) hybrid density functional.^26^ PBE0 has been validated in numerous benchmarking studies for transition-metal chemistry including hydricities of 3d metal hydrides,^27^ heats of formation for large main group compounds,^28^ and structural/thermochemical predictions for 3d transition-metal complexes, nanoclusters and diatomics.^29–31^ These benchmarks support its suitability for modeling copper–mesityl oligomers. However, we also repeated all geometry optimizations and frequency calculations using the MN15 functional to provide an additional reference.^32^ The 6–311+G* basis set was employed for Cu atoms while 6–31G* was used for carbon C and hydrogen H atoms. A similar basis set has been used in previous calculations on copper Cu­(I) systems.^33–36^ All ground-state structures were validated to be at energy minima using frequency calculations at the same level of theory to characterize the nature of the stationary points. The calculations were performed using Gaussian16.^37^ The temperature and pressure used for the thermal corrections are 298.15 K and 1 atm.

Negative (imaginary) frequencies were encountered in some oligomer structures during Gaussian frequency calculations. To resolve this, we displaced the molecule along the vibrational mode corresponding to the imaginary frequency (i.e., the eigenvector of the imaginary frequency) to guide the molecule toward a true minimum on the potential energy surface. We iteratively relaxed the structure until all vibrational frequencies became real. As a result of this refinement, only the monomers and trimers retained their original point group symmetries after optimization. The other optimized oligomers had reduced symmetry: the C_2h_ dimer and C_6h_ hexamer both turned to C_i_, the C_4h_ tetramer became S_4_, and the C_5h_ pentamer and C_7h_ heptamer both gave structures with C_1_ symmetry.

To compare the free energies of formation of the different [CuMes]*_n_* cyclic oligomers from the monomer unit, we computed ΔG using the total free energy of [CuMes]*_n_* divided by *n* and subtracted the free energy of the reference monomer optimized at the same level of theory, as shown in eq 1. The relative enthalpy and entropy contributions to the free energy were computed analogously.

$$\mathrm{\Delta G}=G_{\left[\mathrm{CuMes}\right]n}/n-G_{\mathrm{CuMes}}$$ 1

### Bond Order Analysis

To further probe the bonding characteristics within the CuMes oligomers, we performed quantitative bond order analyses using Multiwfn version 3.8.^38,39^ In addition, natural bond orbital (NBO) analysis^40^ was carried out using NBO version 3.1 as implemented in Gaussian16. This combined approach allowed us to monitor how the strength and character of both Cu^I^–ligand and Cu^I^···Cu^I^ interactions evolved with oligomerization.

### Orbital Composition Analysis

To help explain the relative stabilities of the CuMes oligomers, we carried out an orbital composition analysis focusing on the frontier molecular orbitals. Specifically, we quantified the atomic orbital contributions (*s*, *p*, and/or *d*) of copper, carbon, and hydrogen to the highest [10×*n*] orbitals of [CuMes]*_n_*. These orbitals include all of the π orbitals of the mesityl ligand, the *d* orbitals of the copper, as well as any orbital involved in the Cu^I^···Cu^I^ interactions and Cu^I^–C bonding. The orbital composition analysis was performed using the Multiwfn program^38,39^ using Gaussian16-generated log (.log) files.

## Results and Discussion

Figure 2 displays the relationship between enthalpy (ΔH in red circles), negative entropy contribution to free energy (-TΔ*S*in blue squares), and Gibbs free energy (ΔG in purple crosses) across different oligomer states from monomer to heptamer. As shown in eq 1, these energies are reported per unit CuMes and relative to the monomer unit, which serves as the reference with a relative energy of 0.00 kcal/mol. The top panel reports energies computed with the PBE0 functional while the bottom panel reports energies with the MN15 functional.

**Figure 2:**
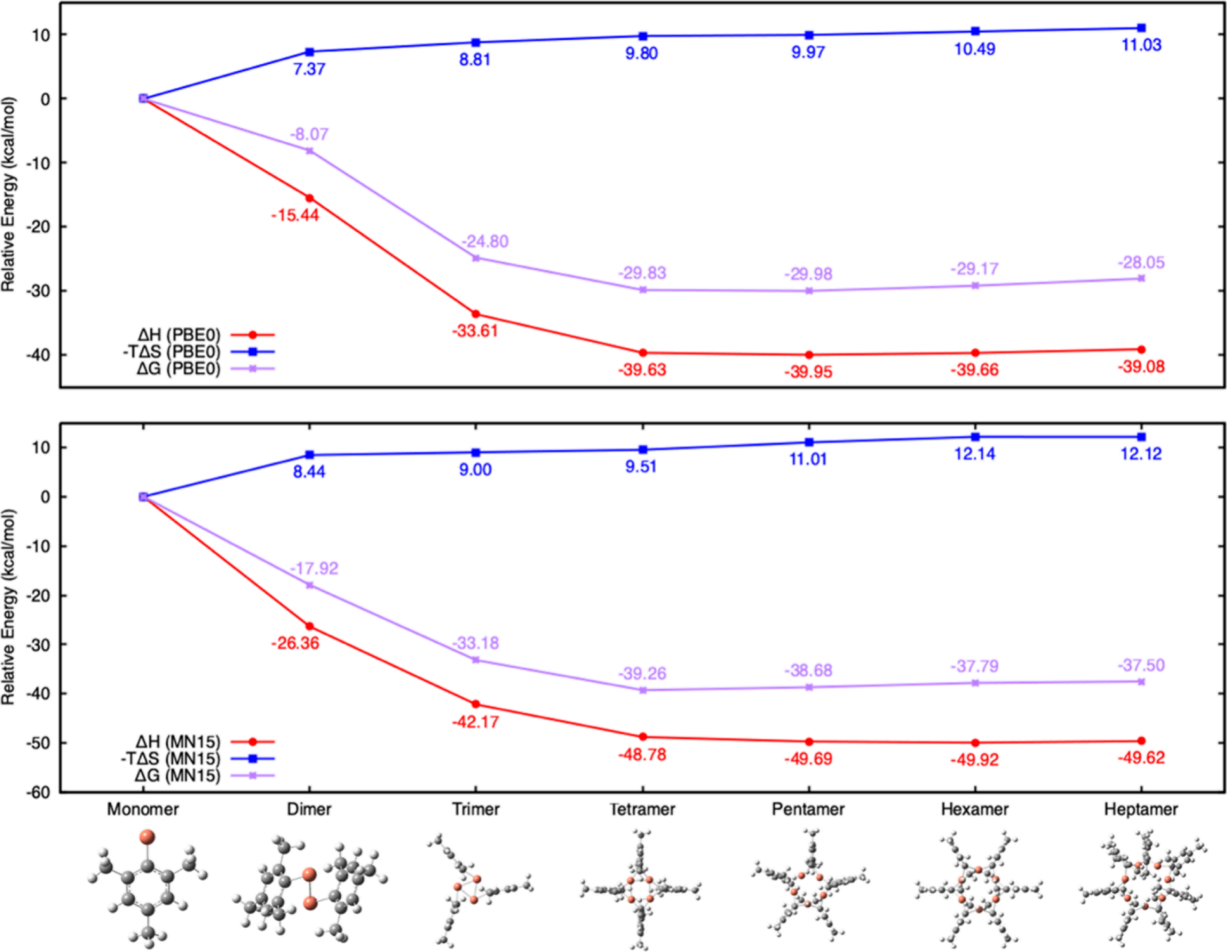
Computed relative ΔH (red circles), -TΔ*S*(blue squares), and ΔG in (purple x’s) of mesitylcopper oligomers compared to the monomer (set to 0.00 kcal/mol). **Top panel**: PBE0 energies; **bottom panel**: MN15 energies. Ball-and-Stick models of the oligomers optimized with the PBE0 functional are shown below the plots.

Both PBE0 and MN15 results indicate that oligomerization is a highly exothermic process. The largest stabilization is observed when moving from the monomer to the dimer and trimer. This trend then stabilizes for larger oligomers (tetramer to heptamer), suggesting that the largest change in bonding character occurs in the smaller oligomers while in larger oligomers the stability is driven by more subtle effects such as steric interactions.

The entropic contribution to free energy, – TΔS, increases gradually with oligomer size consistent with the expectation of lower entropy associated with larger molecular constructs. This entropy is calculated by including the difference between an oligomer and freely moving monomer units, which is more suitable for a gas phase reaction rather than a molecule in solution. The entropy for oligomerization in solution can be estimated more accurately, for instance, by using vibrational entropy obtained quantum mechanically and translational+rotational entropy obtained from molecular dynamics of the monomers and oligomers in solution.^41^ However, here, we expect that the gas phase calculations provide an upper limit for the increase in the – TΔ*S*contribution to free energy with increasing oligomer size, as this entropy change should be smaller in solution.

Figure 2 indicates that all oligomeric forms of mesitylcopper are thermodynamically favored over the monomer. The MN15 functional yields systematically more exothermic enthalpies (∼8–10 kcal/mol relative to PBE0) and correspondingly lower free energies (∼9 kcal/mol). The largest difference is observed when going from the monomer to the dimer, where PBE0 reports an enthalpy change of −15.44 kcal/mol compared to MN15’s −26.36 kcal/mol. PBE0 and MN15 give similar geometries for the monomer, but the dimer geometries optimized at the PBE0 and MN15 levels of theory are different. This is discussed in more detail later in Figure 3. In particular, the PBE0-optimized dimer geometry suggests an additional bonding interaction between the copper atom and the π-system of the mesityl ring, consistent with an asymmetric η^2^-type coordination. This type of coordination is well established for Cu­(I) complexes as illustrated for example in structure III in Figure 1. Beyond the dimer, PBE0 and MN15 give more consistent relative energies for the larger oligomers. Both PBE0 and MN15 locate a free-energy minimum in the tetramer–pentamer range, although there is reversal in the relative ordering: PBE0 slightly favors the pentamer (by ΔG ≈ −0.15 kcal/mol relative to the tetramer), while MN15 slightly favors the tetramer (ΔG ≈ −0.58 kcal/mol relative to the pentamer). Both methods predict the hexamer as the third most stable species.

**Figure 3:**
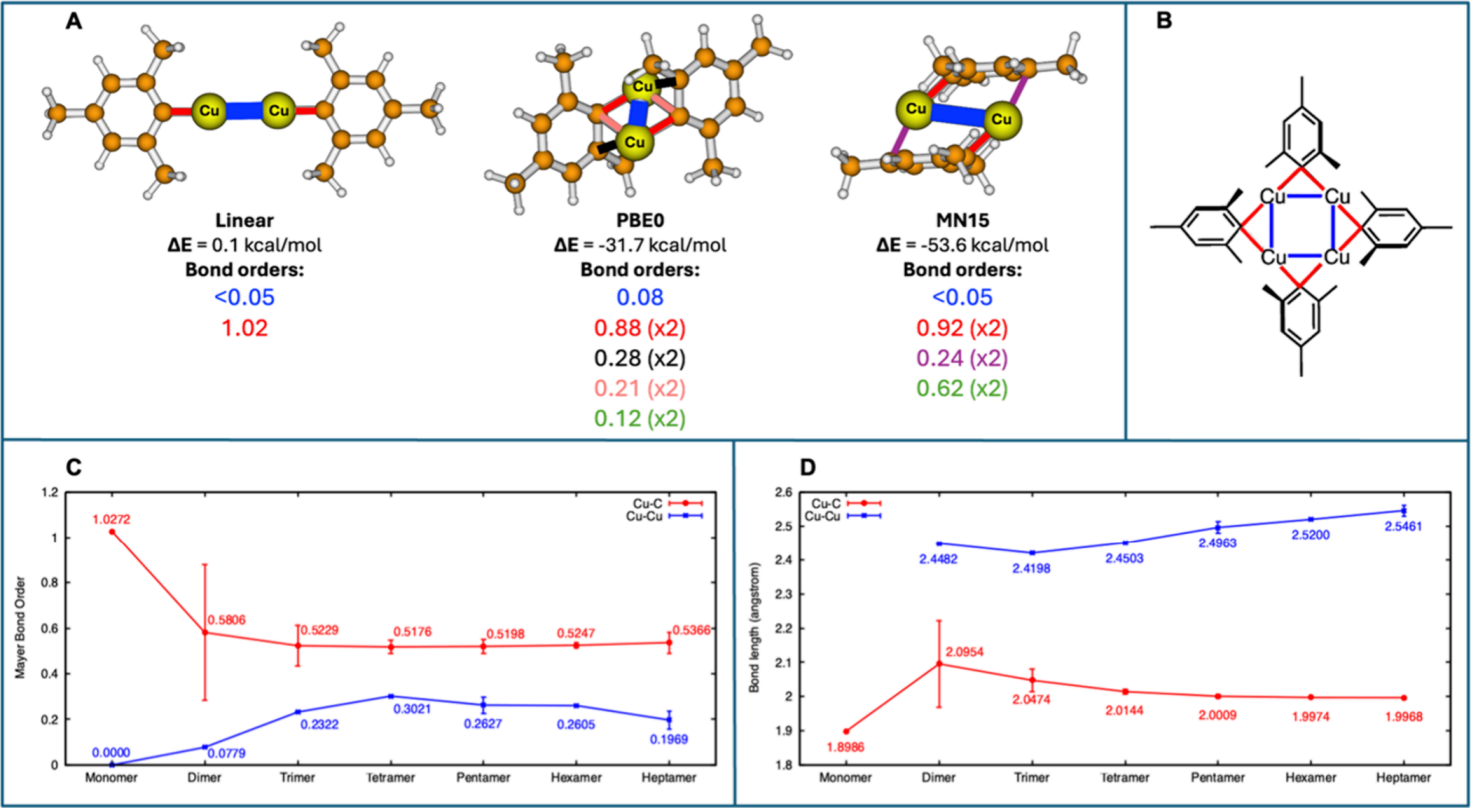
(**A**) Comparison of relative electronic energies (ΔE) and select Cu···Cu and Cu–C Mayer bond orders for three dimer structures. The first geometry is a nonequilibrium linear arrangement, the second structure is a fully optimized dimer with PBE0, and the third structure is the dimer optimized with MN15. The ΔEs are reported relative to the energy of two separate monomers. All Cu–C bond orders larger than 0.2 are labeled explicitly in the figure, while other Cu–C bond orders below 0.2 are summed and indicated in green font. (**B**) A schematic of the tetramer highlighting the representative Cu–C (red) and Cu···Cu (blue) included in the bond order and bond length analyses in panels C and D. (**C**) A plot of *average* PBE0 Mayer bond orders for specific Cu–C (red) and Cu···Cu (blue) bonds. The bonds included in the analysis are ones involved in the μ bridging (i.e., only those highlighted in red and blue in panels A and B). Error bars are used to indicate the range of actual Mayer bond orders. (**D**) average bond lengths of the same Cu–C (red) and Cu···Cu (blue) bonds for [CuMes]*_n_* (*n* = 1–7).

Experimental investigations of mesitylcopper oligomerization have been carried out in solution, most notably in aromatic solvents such as toluene and benzene. Early cryoscopic measurements in toluene-d_8_ suggested a dimer as the dominant species, with a minor presence of pentamer (5–6%).^10^ Notably, the interconversion between these species is solvent-dependent: equilibrium is rapidly established for [AgMes]*_n_* and [AuMes]*_n_*, but is slow in the case of [CuMes]*_n_* where the half-life for interconversion can reach up to 56 h in cyclohexane-d_12_. However, subsequent studies that rigorously excluded solvent interference-by recrystallizing from benzene and drying under vacuum-yielded a more reliable aggregation number of 4.16, indicating that the tetramer is the predominant species in solution, with some pentamer present.^12^ These findings are further supported by X-ray crystallography, which confirm the existence of both tetrameric and pentameric forms, with the tetramer being favored under ambient conditions and the pentamer stabilized only below −20 °C.^4^ The close energetic proximity between the tetramer and pentamer in our gas-phase calculations explains their coexistence in solution.^3^ The MN15 functional appears to predict the correct energy ordering in the gas phase (i.e., finding that the tetramer is more stable), while PBE0 predicts a slightly more stable pentamer. We note that the previous density functional theory (DFT) study on CuPh models by Belanzoni et al. have reported similar trends, finding the tetramer and pentamer to be nearly isoenergetic and both significantly more stable than the dimer.^25^

In Figure 3A, we present dimer geometries optimized at the PBE0 and MN15 levels of theory. Those are compared to a dimer optimized at the PBE0 level of theory with symmetry constraints to keep the C–Cu–Cu-C arrangement linear. Below each structure, we report the electronic energy (ΔE) relative to twice the monomer energy computed at the same level of theory. We also report Mayer bond orders for each geometry.^42–44^ We find that a linear geometry does not result in any Cu–Cu bonding, as reflected by the limited change in energy compared to two separate monomers (ΔE = 0.1 kcal/mol) and a bond order <0.05 (0.05 is the minimum print threshold used by default in Multiwfn). Instead, an unconstrained optimization at the PBE0 level of theory results in a geometry where the mesityl carbon bridges the two copper atoms. The bond is not shared equally with the two copper centers; each mesityl group maintains a strong bond with one copper center (bond order 0.88, shown in red) while forming a weak bond with the other copper (bond order 0.21, shown in salmon). At the same time, each mesityl is oriented such that one of its other carbon atoms also forms a partial bond with the copper centers (0.28 bond order, shown in black). Cu–C bonds other than those explicitly highlighted contribute only slightly to the total Cu–C bond order (0.12 for each mesityl, shown in green font). Together, all Cu–C bonds contribute a bond order of 1.49 per mesityl unit, which is considerably higher than the 1.02 in the linear nonbonded dimer. This increase in Cu–C bond order is accompanied by a small increase in the Cu···Cu bond order (0.08 bond order shown in blue). Together, these changes in bonding contribute to a stabilization energy of −31.7 kcal/mol relative to the separate monomers.

In the MN15 calculations, the nature of the bonding interactions between the copper and mesityl moieties differs from the interactions observed with the PBE0 functional; the mesityl still forms a strong bond to one of the copper centers (0.92 bond order, shown in red). However, the next strongest Cu–C bond involves the carbon para to the strongly bonded one (0.24 bond order, shown in purple). Therefore, with MN15, the Cu is situated above the center of the mesityl aromatic group such that copper can form bonding interactions with all aromatic carbons of the mesityl ligand. Other Cu–C bonds involving the mesityl aromatic carbons contribute significantly to the bond order (0.62 bond order, shown in green font). In total, the bond order for the MN15 geometry is 1.78, which is consistent with the larger stabilization of the dimer for MN15.

While the dimer geometry and bonding differ significantly for PBE0 and MN15, the two methods give more consistent geometries and relative energies for larger oligomers. In other words, differences between PBE0 and MN15 are related to the description of the high energy unstable monomer and dimer units. Hereafter, we focus on the PBE0 calculations for analysis.

PBE0 Mayer bond analysis for other oligomers is summarized in Figure 3C. The plots display average bond orders specifically for Cu–C and Cu···Cu bonding interactions involved in the μ-bridging (red and blue lines, respectively, as shown in the example in Figure 3B).^42–44^ The corresponding Wiberg bond orders are shown in the Supporting Information (SI) Figure S1. While Wiberg uses a simpler bond order definition, the Mayer definition is more compatible for molecular orbitals constructed with nonorthogonal basis sets and has found more general applicability to systems including inorganic molecules.^45^ For the Cu–C bond, both Wiberg and Mayer indices indicate a single bond (bond order close to 1) in the monomer, which weakens markedly upon dimerization and remains small in larger oligomers. Specifically, the Mayer bond order for Cu–C starts at approximately 1.03 in the monomer and drops sharply to ∼ 0.58 for the dimer. Beyond the dimer, the Cu–C Mayer bond order remains relatively stable, fluctuating only slightly between 0.52 and 0.54 from the trimer to the heptamer.

The error bars in Figure 3C indicate the range of bond orders for the individual Cu–C and Cu···Cu bonding interactions. Only the dimer displays a large asymmetry in the Cu–C bonds, as already explained in Figure 3A (one bond with 0.88 bond order and one with 0.21 bond order). After the trimer, the interaction becomes more consistent with a regular μ-bridging, where each mesityl unit bridges two adjacent copper atoms with an almost equal strength.

The Cu···Cu bond order displays the opposite trend. The bond order is set by default to 0 in the monomer where there is no Cu···Cu interaction. Upon oligomerization, the Mayer bond order for Cu–Cu rises to 0.08 for the dimer and fluctuates around 0.2–0.3 in larger clusters (Figure 3C). The maximum Cu···Cu bond order is observed for the tetramer (0.30), and the next largest bond order for the pentamer (0.26).

A plot of the average Cu–C and Cu···Cu bond lengths (Figure 3D) indicates a loosely inverse relationship between Mayer bond order and bond length. The Cu–C bond length increases in the dimer relative to the monomer before contracting and stabilizing near 2.0 Å for larger oligomers. Conversely, the Cu···Cu distances grow steadily with increasing *n*, consistent with a corresponding decrease in Mayer bond order from the tetramer onward. This decrease in Cu···Cu bond order after the tetramer (and corresponding increase in Cu···Cu bond distance) may be associated with steric interactions between the mesityl units in larger oligomers, as discussed in more detail later.

Taken together, these trends in bond order explain the driving forces for oligomerization. In the dimer, the single Cu–C bond in each monomer is replaced by a weak bridging interaction, as shown in Figure 3A and the associated discussion. The further stabilization for the trimer can be rationalized not only by a more symmetric μ-bridging interaction (i.e., the formation of two bonds by each mesityl with, on average, a bond order of 0.52 each) but also by the fact that the trimer introduces two new Cu···Cu contacts with a bond order of 0.23 each. This is in contrast to subsequent oligomers, which each produce only one Cu···Cu contact with each expansion of the ring, and where therefore energetic changes are more subtle.

Figure 4 displays the ten highest occupied molecular orbitals (MOs) for the monomer. The choice to focus on those 10 MOs is based on the orbital energies plotted in SI Figure S2, where the monomer and oligomers orbitals in the range of −25 to 0 eV are shown. Some of those orbitals can be easily assigned visually. For instance, HOMO–3 and HOMO–4 of the monomer clearly display Cu­(*d*) character. On the other hand, some orbitals like HOMO–6 and HOMO–7 have mixed character. Several of those orbitals have electron density between the Cu and mesityl carbon and can explain the bonding character in the monomer; The HOMO–1 shows an *s*-like orbital on Cu that appears clearly polarized toward the mesityl group. HOMO–6, HOMO–7, HOMO–8, and HOMO–9 all show some bonding character between the *d* orbitals of the Cu and *p* orbitals of the mesityl group. However, the same visual inspection and orbital assignments become more complicated when moving to larger oligomers. Therefore, we employed Multiwfn to quantify the mixing by computing orbital contributions from Cu­(*s*), Cu­(*p*), Cu­(*d*), C­(*s*), C­(*p*), H­(*s*), and a collective “Other” category. The “Other” component accounts for electron density not clearly attributable to those six atomic orbitals. The results are shown in Figure 5 where Cu orbitals are depicted in different shades of yellow/gold, carbon orbitals in different shades of gray, H(s) orbitals in light gray, and other orbitals in navy. The vertical axes in Figure 5 represent the percent contribution of each atomic orbital, while the horizontal axes correspond to the orbital number, starting from the HOMO (orbital 1).

**Figure 4:**
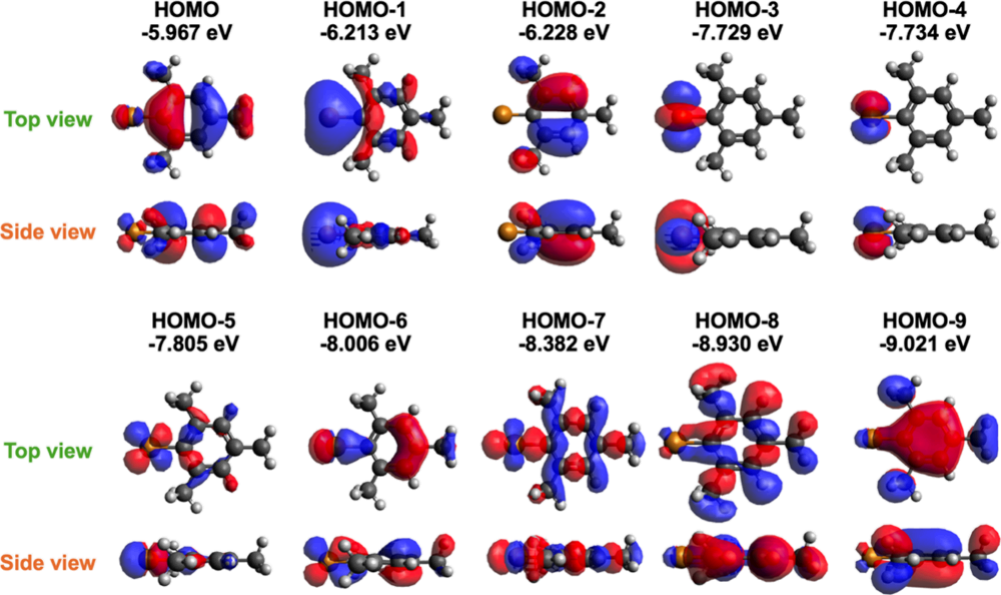
Top and side views of the ten highest occupied molecular orbitals (HOMO to HOMO–9) of the monomer with their energies.

**Figure 5:**
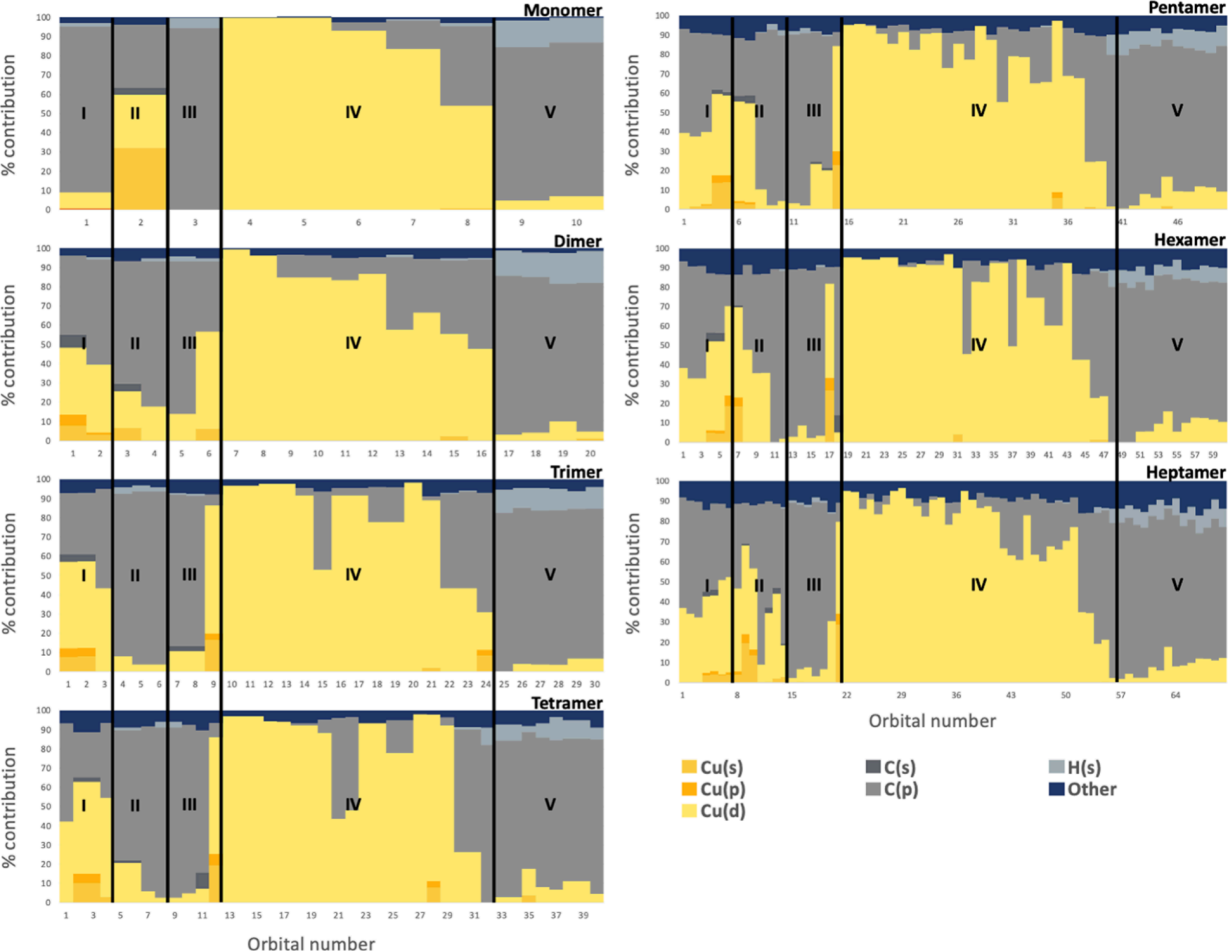
Stacked bar plots showing the atomic orbital composition of occupied molecular orbitals in [CuMes]*_n_* from *n* = 1–7 (see title on top right of each plot). The vertical axis indicates percent contributions (%) of atomic orbitals, and the horizontal axis denotes orbital index starting from the HOMO (labeled as 1). The legend shows the color-coding based on orbital contributions. The orbitals are divided into five regions: I, II, III, IV, and V, scaled proportionally to *n* (region I: 1*n*, region II: 1*n*, region III: 1*n*, region IV: 5*n*, region V: 2*n* orbitals). These subdivisions facilitate the comparison of the orbital character across oligomers.

Because the number of orbitals within the energy window varies depends on *n* for [CuMes]*_n_*, we divided the range into five discrete regions (I–V) to allow meaningful comparison (Figure 5). Each region contains a multiple of *n* orbitals: region I contains 1*n*, region II: 1*n*, region III: 1*n*, region IV: 5*n*, and region V: 2*n* orbitals. For example, region I includes one orbital (HOMO) for the monomer, two orbitals for the dimer (HOMO, HOMO–1), and three orbitals for the trimer (HOMO to HOMO–2), and so on, while region IV contains five orbitals for the monomer, ten orbitals for the dimer, etc.

To interpret the plots in Figure 5, it is helpful to begin with the monomer (top left) and to compare its orbital composition to the corresponding molecular orbitals shown in Figure 4. According to Figure 5, the HOMO of the monomer (labeled as orbital 1) is primarily composed of carbon *p* orbitals, with minor contributions from copper *d* orbitals, indicating that this is a π orbital localized on the ligand as shown in Figure 4 for the HOMO. In the monomer’s HOMO–1 (orbital 2), there is an increased contribution from copper *p* and carbon *s* orbitals, which aligns with the Cu–C sigma bonding character seen in Figure 4. The monomer’s HOMO–2 is a ligand-centered π orbital, dominated by C­(*p*). The next five orbitals (HOMO–3 to HOMO–7 in Figure 4, corresponding to orbitals 4–8 in the monomer plot in Figure 5) are all predominantly Cu­(*d*) orbitals. However, we also see clearly mixing in of C­(*p*) character in some of those orbitals, consistent with the π-orbital mixing observed in Figure 4.

As shown in Figure 5, region I in the monomer is composed almost entirely of π-type orbitals. However, upon oligomer formation, this region begins to mix significantly with Cu­(*s*), Cu­(*p*), Cu­(*d*), and C­(*s*) orbitals. As a result, two orbital populations are altered significantly during oligomerization: **region I** and **region II**. These electronic redistributions are the ones responsible for most of the bonding character changes detected in the bond order plots in Figure 3. To quantify these changes, we averaged orbital compositions within region I and II for all oligomers and plotted them in Figure 6 (the corresponding data is provided in Figures S4–S5). In the monomer, region I is dominated by π-type orbitals localized on the mesityl ligand, with C­(*p*) contributing 86.2% of the character. This strong ligand-centered bonding is accompanied by only minor contributions from copper-based orbitals, such as Cu(s) and Cu­(d). Upon oligomerization, the C­(*p*) contribution in region I drops significantly, staying around 34 – 43% for the tetramer and pentamer, while Cu­(*d*) character increases substantially—rising from negligible levels in the monomer to a peak of 47.4% in the tetramer and remaining high in the pentamer at 39.3%. This shift marks a transition from localized ligand-based bonding to a more delocalized and cooperative bonding involving metal–metal interactions.

**Figure 6:**
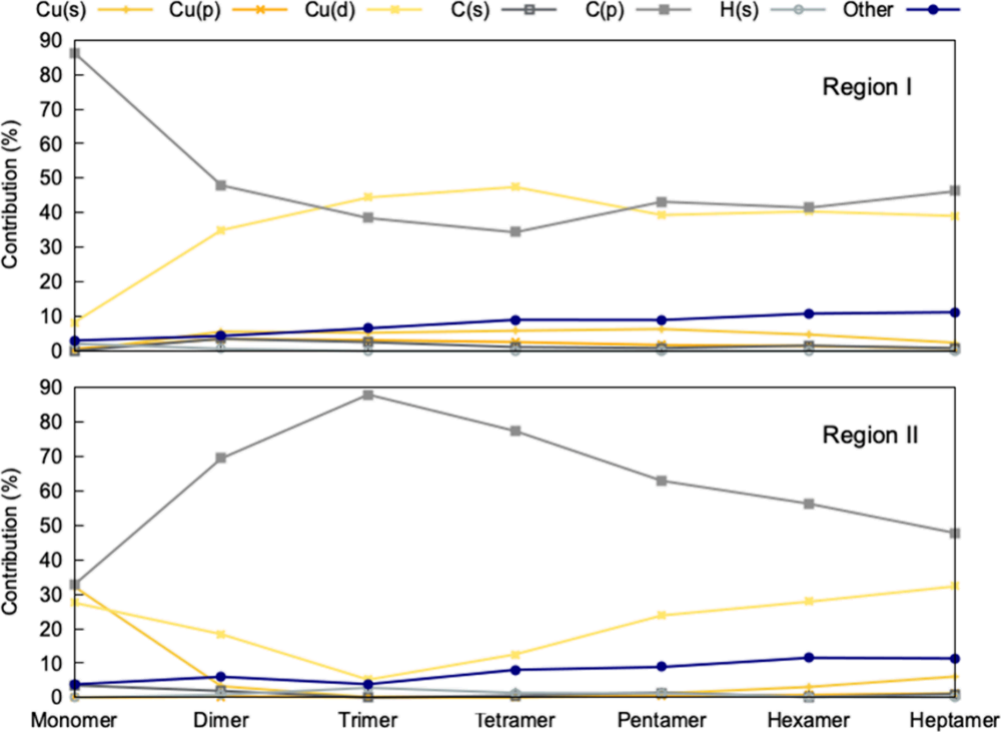
Average orbital compositions in region I (top) and region II (bottom) across increasing mesitylcopper oligomer size from monomer to heptamer. Values represent the average percent contribution of each atomic orbital type—Cu­(*s*), Cu­(*p*), Cu­(*d*), C­(*s*), C­(*p*), H­(*s*), and Other—where ″Other″ includes unassigned or delocalized electron density.

Region II displays a complementary redistribution of orbital character. In the monomer, this region, corresponding to the HOMO–1 orbital, comprises a mix of C­(*p*), Cu­(*s*), and Cu­(*d*) character and is largely responsible for the Cu–C bonding character in the monomer. Oligomerization leads to a large increase in C­(*p*) character in the trimer, followed by a gradual decline in larger clusters. Cu­(*s*) character, which is quite dominant in the monomer, is effectively lost in all the oligomers, which is associated with the drop in Cu–C bond order in Figure 3. Cu­(*d*) contributions in Region II show a U-shaped trend complementary to the C­(*p*) character, indicating a shift to/from a more ligand centered orbital in the trimer compared to the other oligomers where the orbital is more delocalized.

Regions III–V also undergo some changes in character, but they are not as dramatic as in regions I and II (see SI Figures S3 and S6–S8). Orbitals in region III, originally another π bond in the monomer, mostly retain their π character across all oligomers, although there is Cu­(*d*) character mixing in from region IV. This mixing is most clearly seen in the dimer and trimer, but then decreases with larger oligomers (Figure S6). Correspondingly, the orbitals in region IV, which originally are predominantly Cu­(*d*) character, gain a small degree of C­(*p*) character (Figure S7). Region V maintains its ligand-centered character but has a small extent of growing Cu­(*d*) and other contributions with increasing oligomer size (Figure S8).

In addition to electronic effects on cluster stability, steric effects may also contribute to the minor variations in relative cluster stability, especially in the larger oligomers. The evolution of key bond angles—Cu–C–Cu (θ_1_), C–Cu–C (θ_2_), and Cu–Cu–Cu (θ_3_)—across the mesitylcopper oligomer series are shown in Figure 7. The Cu–C–Cu θ_1_, the angle at the bridging carbon of the mesityl ligand, increases modestly from 70.23° in the dimer to 79.22° in the heptamer. This widening may help reduce steric effects on the mesityl carbon donor atom with increasing oligomer size. However, this effect is likely to be small, especially beyond the tetramer.

**Figure 7:**
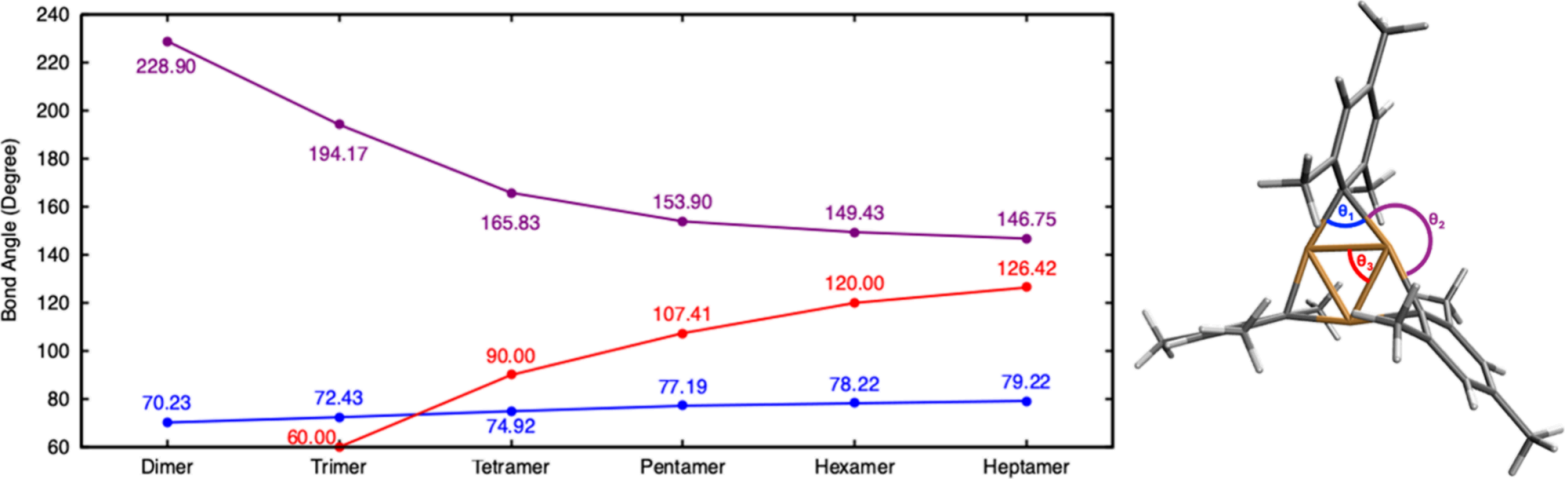
Variation in Cu–C–Cu (θ_1_, blue), C–Cu–C (θ_2_, purple), and Cu–Cu–Cu (θ_3_, red) bond angles for mesitylcopper oligomers optimized at the PBE0 level of theory. The three angles are labeled for the trimer structure on the right.

The other two angles (θ_2_ and θ_3_), which are centered on the copper atoms, show more significant changes. The θ_2_ angle represents the geometry on the outer side of the copper­(I) atoms. This angle is a concave angle (>180°) for the dimer and trimer but becomes convex starting from the tetramer. Conversely, the Cu–Cu–Cu θ_3_ angles gradually increase from 60° in the trimer to 126.42° in the heptamer. These values closely track the internal angles of ideal polygons (triangle: 60°, square: 90°, pentagon: 108°, hexagon: 120°, heptagon: 128.6°). The large θ_2_ and small θ_3_ angles here represent an unusual bonding geometry for copper­(I) complexes, especially in the smaller oligomers (trimer and tetramer) where each Cu­(I) is surrounded by two mesityl carbon atom donors and two other copper atoms in a “flattened seesaw” arrangement. This close arrangement of surrounding groups likely results in steric strain. While this steric strain is released when moving to larger oligomers, it will be offset by the reduction in θ_3_ which brings the bulky mesityl groups closer together; a smaller θ_3_ will result in destabilizing steric effects from the methyl groups on the different mesityl clashing together (see also the space-filling models in Figure S9). Such steric effects contribute to deviations from planarity, which is most noticeable in the hexamer and heptamer. The pentamer geometry, which has internal angles large enough to relieve the acute angle strain and steric congestion of smaller rings while still having the mesityl groups well separated (153.9°), likely explains its relative thermodynamic stability relative to other oligomers. To a lesser extent, the same applies to the tetramer which has the mesityl groups the further apart (165.83°) although at the cost of some additional internal structural strain.

Similar bending distortions of the μ-mesityl ligand have also been observed in dicopper­(I) complexes supported by proton-responsive PNNP-type pincer ligands, as reported by Broere and co-workers.^20^ In that work, changes in ligand protonation state led to significant bending (Cu–C–C angles) and tilting of the μ-mesityl ligand relative to the dicopper plane. While our analysis focuses on Cu–C–Cu (θ_1_), C–Cu–C (θ_2_), and Cu–Cu–Cu (θ_3_) angles in neutral oligomers, both studies highlight that μ-mesityl bridges accommodate electronic/steric demands through geometric distortion. The results in Figure 7 highlight the subtle balance between angle strain relief and methyl–methyl repulsion that contributes to the nonmonotonic stability trend observed in the oligomer series.

## Conclusion

Mesitylcopper oligomerization is driven by a significant electronic stabilization, but the optimal size of the oligomer is determined by a delicate balance between enthalpic stabilization and entropic penalties. The electronic stability arises from enhanced Cu···Cu bonding interactions and cooperative Cu­(*d*)-C­(*p*) interactions in midsized clusters, as evidenced by bond order indices and orbital composition analyses. These calculations indicate a strong electronic driving force at least for formation of a trimer cyclic oligomer. For the dimer, this driving force is related to formation of an asymmetric μ-bridging interaction between the mesityl ligands and two copper centers that only moderately increases the Cu···Cu bond order but introduces a large increase in total Cu–C bond order. This bonding interaction, however, is described differently by the two functionals tested (PBE0 and MN15). The trimer stabilization is associated with a more symmetric μ-bridging that introduces two new Cu···Cu bonding interactions to the dimer structure. Beyond the trimer, however, the differences in energies between the oligomers are more nuanced, such that both entropic and structural (steric) considerations start to become relevant. Specifically, angle strain of the copper­(I) cyclic core can energetically disfavor the smaller oligomers such as the trimer while both entropy and steric interactions of the bulky μ-bridging mesityl groups disfavor the larger oligomers such as the heptamer. Gas-phase calculations predict the pentameric form as the most stable oligomer, with the tetramer and hexamer closely following.

The computational results presented here can provide guidelines for how to tune the structure and reactivity of organocopper reagents and catalysts. The ability to stabilize homoleptic Cu^I^_2_(aryl) dimers (e.g., see **III** in Figure 1)^15^ and heteroleptic Cu^I^_3_ trimers with sterically demanding ligands^19^ is one such example where using tailored substituents has been shown to modulate aggregation states for targeted reactivity.

## Supplementary Material



## Notes

Average Wiberg bond orders of Cu–C and Cu···Cu across oligomers, orbital energies versus density of states across oligomers, average orbital compositions in five regions across oligomers, space-filling models of the oligomers, coordinates of the monomer and oligomers optimized at the PBE0 and MN15 levels of theory (PDF)
