# Supplementary material for: How Thermodynamic, Electronic, and Steric Factors Influence Mesitylcopper Oligomers
Source: J Phys Chem A. 2025 Oct 24;129(44):10091–100. doi: 10.1021/acs.jpca.5c04666 (PMC12598852; doi:10.1021/acs.jpca.5c04666)
Supplement: Supplementary file 1 [file jp5c04666_si_001.pdf]

Supporting information for:

How Thermodynamic, Electronic, and Steric Factors Influence Mesitylcopper Oligomers

D.P. Ngan Le,[†] Michael Stollenz,[§] Samer Gozem*[,†]

[†] Department of Chemistry, Georgia State University, Atlanta, Georgia 30303, United States

[§] Department of Chemistry and Biochemistry, Kennesaw State University, Kennesaw, Georgia 30144, United States

Table of content

Figure S1: Average Wiberg bond orders of Cu-C and Cu···Cu across oligomers.

Figure S2: Orbital energies versus density of states across oligomers.

Figure S3: Average orbital compositions in five regions across oligomers.

Figure S4: Average orbital compositions in region I across oligomers.

Figure S5: Average orbital compositions in region II across oligomers.

Figure S6: Average orbital compositions in region III across oligomers.

Figure S7: Average orbital compositions in region IV across oligomers.

Figure S8: Average orbital compositions in region V across oligomers.

Figure S9: Space-filling models of the optimized oligomers.

Initial XYZ coordinates of monomer.

PBE0-optimized XYZ coordinates of mesitylcopper from monomer to heptamer.

MN15-optimized XYZ coordinates of mesitylcopper from monomer to heptamer.

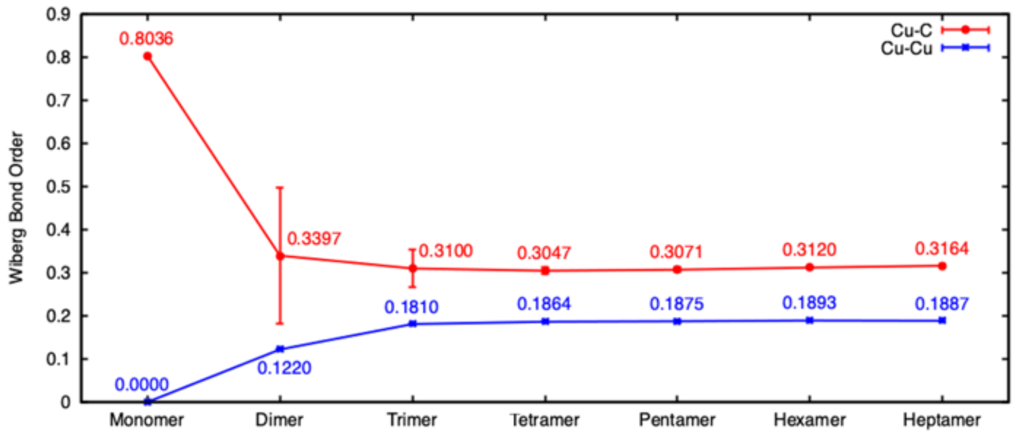

Figure S1. Average Wiberg bond orders of adjacent Cu-C (red) and Cu···Cu (blue) bonds for $[CuMes]_n$ (n=1-7). Error bars reflect standard deviations across equivalent bonds in each oligomer.

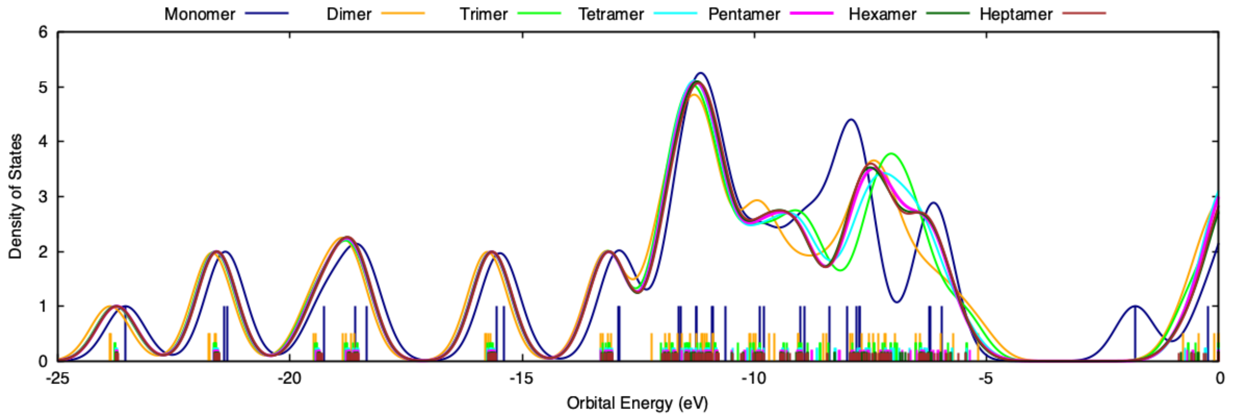

Figure S2. PBE0 MO energy distribution (in eV) with density of states (DOS) for $[CuMes]_n$ (n=1-7). Energies from individual calculations are indicated as impulse lines in navy (n=1), orange (n=2), green (n=3), cyan (n=4), magenta (n=5), dark green (n=6), and brown (n=7). These lines were convolved with 1 eV wide (FWHM) Gaussian functions which were summed to give the final computed "spectrum" of density of states. By scaling the number density by the number of units in the oligomer, n, the convolved density of states helps reveal changes in electronic structure arising from degeneracy breaking (which leads to a widening of the convolved bands) or shifting of energies. We found that the electronic structure of the monomer is most distinct from the remaining oligomers. We also found that most changes in the orbital energies upon oligomerization occur in the range of -10 to -5 eV. This energy range includes the ten highest molecular orbitals of the monomer which are analyzed in more detail in the main text.

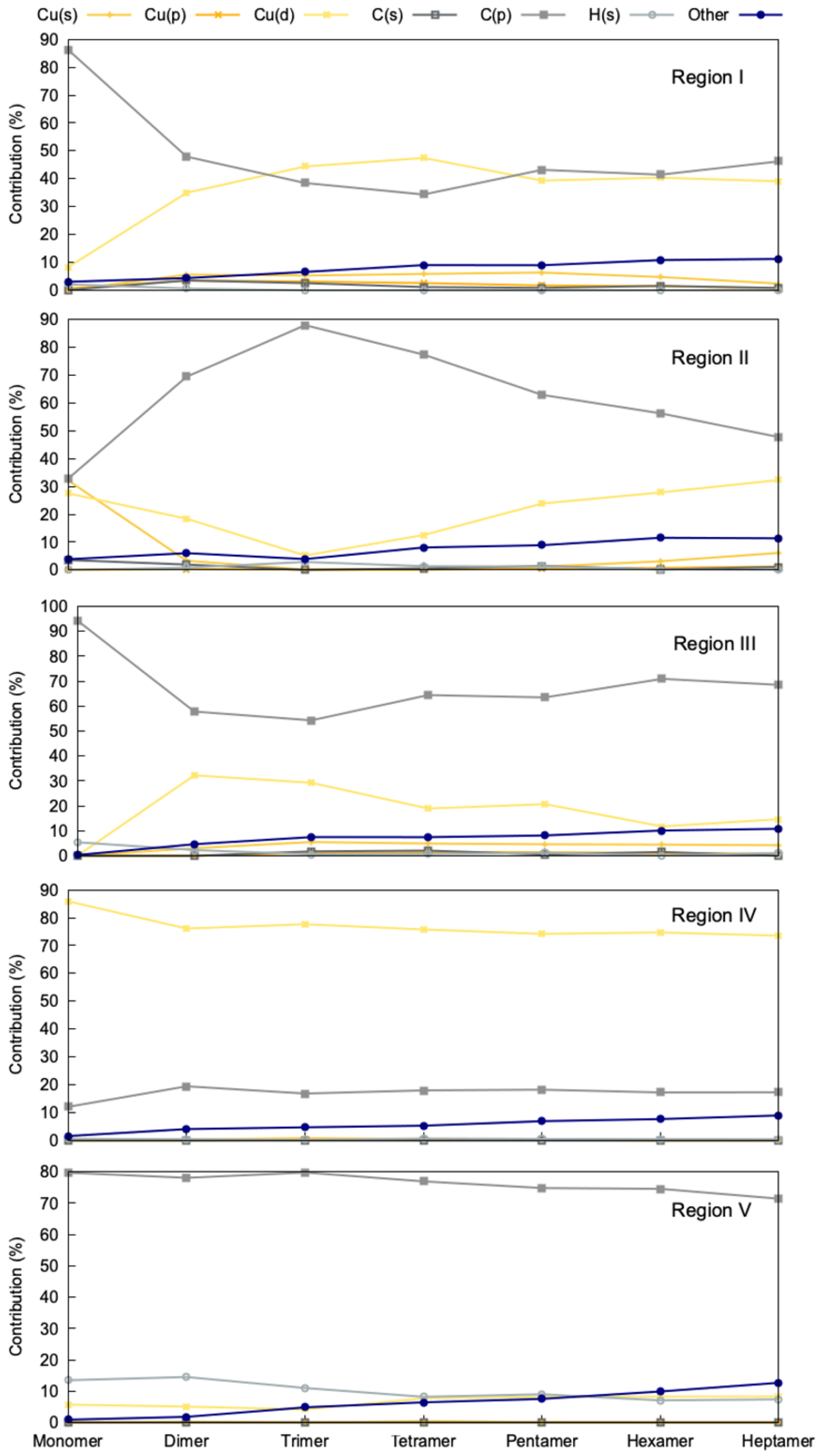

Figure S3: **Average orbital compositions in five regions (I-V) across increasing mesitylcopper oligomer size from monomer to heptamer**. Values represent the average percentage contribution of each atomic orbital type—Cu(s), Cu(p), Cu(d), C(s), C(p), H(s), and Other—where "Other" includes unassigned or delocalized electron density.

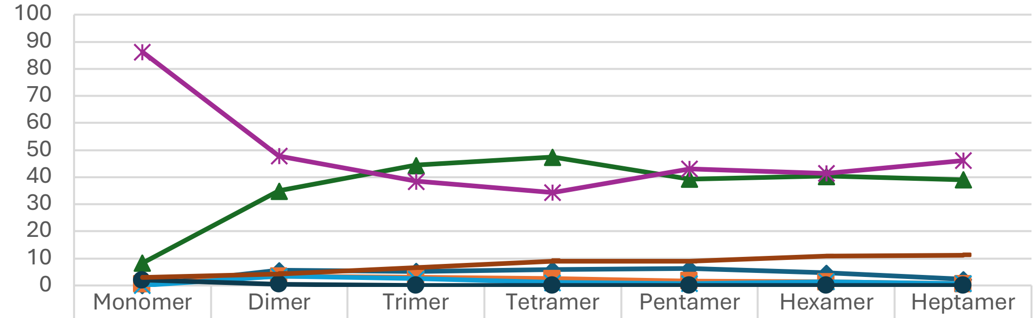

	Monomer	Dimer	Trimer	Tetramer	Pentamer	Hexamer	Heptamer
Cu(s)	0.000	5.528	5.186	5.788	6.275	4.670	2.286
Cu(p)	0.540	3.412	3.059	2.489	1.642	1.369	0.610
Cu(d)	8.251	34.924	44.373	47.417	39.297	40.330	39.027
C(s)	0.000	3.437	2.475	1.053	0.741	1.477	0.724
C(p)	86.233	47.881	38.420	34.305	43.148	41.411	46.204
H(s)	2.022	0.534	0.000	0.000	0.000	0.000	0.000
Other	2.954	4.284	6.487	8.948	8.897	10.743	11.149

Figure S4: **Average orbital compositions in region I across increasing mesitylcopper oligomer size from monomer to heptamer**. Values represent the average percentage contribution of each atomic orbital type—Cu(s), Cu(p), Cu(d), C(s), C(p), H(s), and Other—where "Other" includes unassigned or delocalized electron density.

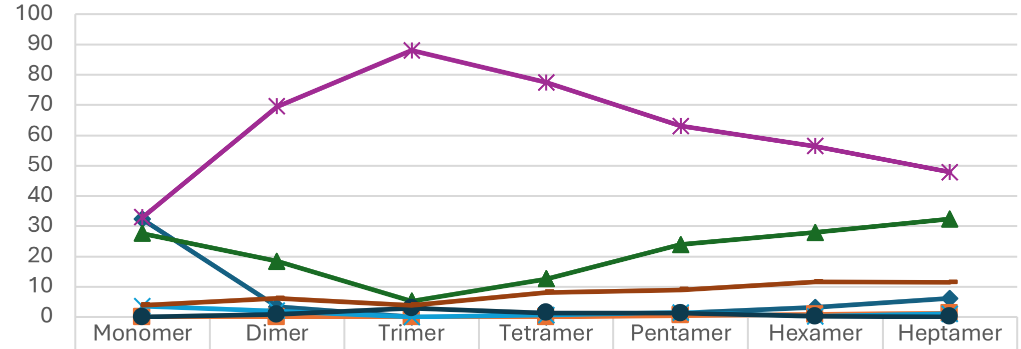

	Monomer	Dimer	Trimer	Tetramer	Pentamer	Hexamer	Heptamer
Cu(s)	32.194	3.301	0.000	0.000	1.191	3.101	6.136
Cu(p)	0.000	0.000	0.000	0.000	0.439	0.754	1.231
Cu(d)	27.535	18.409	5.200	12.556	23.879	27.865	32.354
C(s)	3.565	1.935	0.000	0.572	1.401	0.189	1.051
C(p)	32.862	69.502	88.031	77.451	62.976	56.334	47.771
H(s)	0.000	0.810	2.877	1.353	1.220	0.193	0.086
Other	3.844	6.043	3.893	8.069	8.895	11.564	11.371

Figure S5: Average orbital compositions in region II across increasing mesitylcopper oligomer size from monomer to heptamer. Values represent the average percentage contribution of each atomic orbital type—Cu(s), Cu(p), Cu(d), C(s), C(p), H(s), and Other—where "Other" includes unassigned or delocalized electron density.

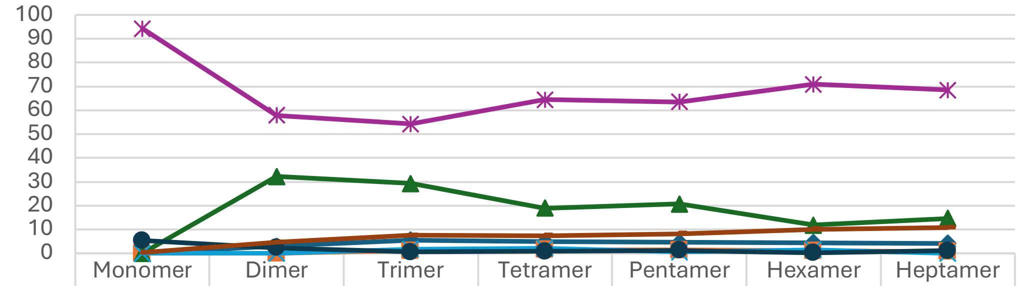

	Monomer	Dimer	Trimer	Tetramer	Pentamer	Hexamer	Heptamer
Cu(s)	0.000	3.031	5.485	4.897	4.594	4.466	4.210
Cu(p)	0.000	0.000	1.137	1.447	1.402	1.056	0.749
Cu(d)	0.000	32.250	29.260	18.937	20.692	11.759	14.636
C(s)	0.000	0.000	1.748	2.079	0.515	1.509	0.000
C(p)	94.300	57.822	54.292	64.415	63.482	70.919	68.513
H(s)	5.392	2.267	0.598	0.768	1.170	0.184	1.090
Other	0.308	4.630	7.481	7.458	8.145	10.107	10.802

Figure S6: Average orbital compositions in region III across increasing mesitylcopper oligomer size from monomer to heptamer. Values represent the average percentage contribution of each atomic orbital type—Cu(s), Cu(p), Cu(d), C(s), C(p), H(s), and Other—where "Other" includes unassigned or delocalized electron density.

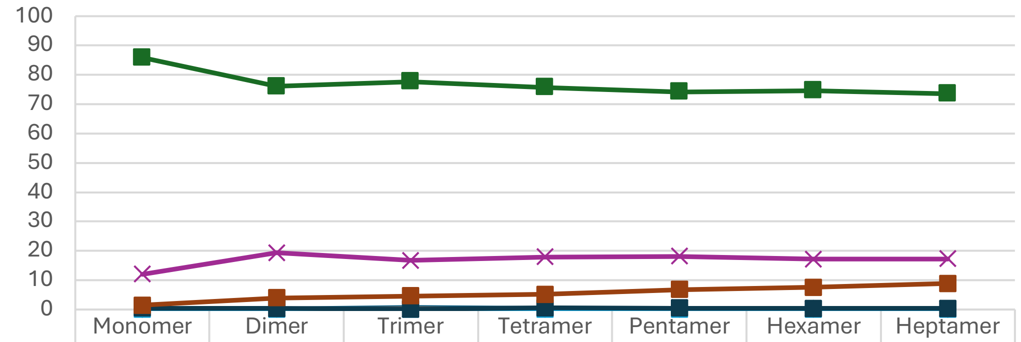

	Monomer	Dimer	Trimer	Tetramer	Pentamer	Hexamer	Heptamer
Cu(s)	0.152	0.234	0.711	0.391	0.310	0.211	0.017
Cu(p)	0.000	0.000	0.186	0.170	0.120	0.000	0.000
Cu(d)	85.873	76.091	77.649	75.750	74.174	74.697	73.539
C(s)	0.000	0.000	0.000	0.000	0.000	0.000	0.000
C(p)	11.994	19.362	16.765	17.896	18.146	17.158	17.210
H(s)	0.489	0.347	0.084	0.590	0.432	0.362	0.351
Other	1.493	3.967	4.605	5.202	6.818	7.572	8.883

Figure S7: Average orbital compositions in region IV across increasing mesitylcopper oligomer size from monomer to heptamer. Values represent the average percentage contribution of each atomic orbital type—Cu(s), Cu(p), Cu(d), C(s), C(p), H(s), and Other—where "Other" includes unassigned or delocalized electron density.

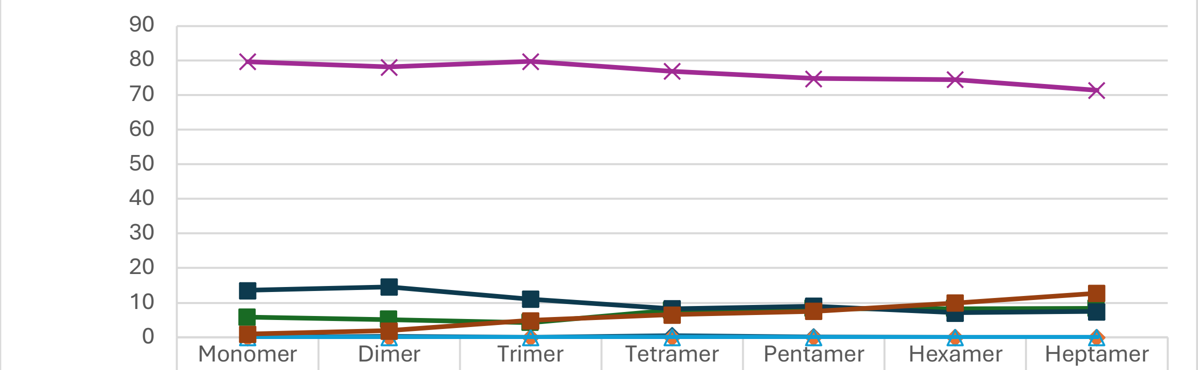

	Monomer	Dimer	Trimer	Tetramer	Pentamer	Hexamer	Heptamer
Cu(s)	0.000	0.313	0.000	0.490	0.168	0.000	0.000
Cu(p)	0.000	0.000	0.000	0.000	0.000	0.000	0.000
Cu(d)	5.761	5.129	4.238	7.784	8.333	8.310	8.374
C(s)	0.000	0.000	0.000	0.000	0.000	0.000	0.000
C(p)	79.738	78.125	79.814	76.981	74.849	74.597	71.450
H(s)	13.552	14.572	11.055	8.246	9.058	7.131	7.440
Other	0.950	1.862	4.894	6.500	7.593	9.963	12.736

Figure S8: Average orbital compositions in region V across increasing mesitylcopper oligomer size from monomer to heptamer. Values represent the average percentage contribution of each atomic orbital type—Cu(s), Cu(p), Cu(d), C(s), C(p), H(s), and Other—where "Other" includes unassigned or delocalized electron density.

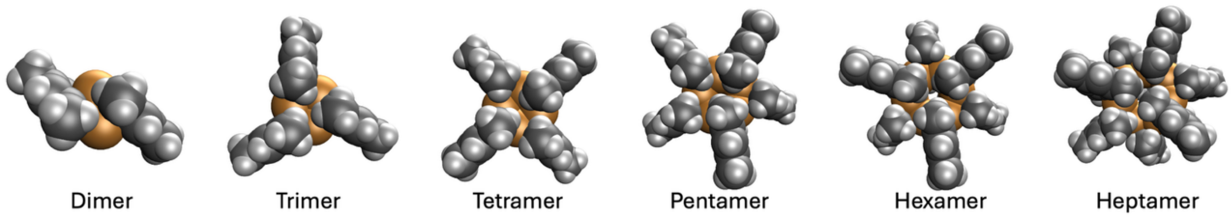

Figure S9. Space-filling models of the optimized oligomers (dimer to heptamer)

Coordinates for initial monomer

Cu	0.0000000	0.0000000	0.0000000
C	0.0000000	3.6614114	-1.2108854
C	0.0000000	4.3583967	0.0000000
C	0.0000000	3.6614114	1.210885
C	-0.0000001	2.2588451	1.2188720
C	0.0000000	1.5600000	0.0000000
C	-0.0000001	2.2588451	-1.2188720
H	0.0000000	4.2136530	-2.1427012
C	0.0000000	5.8583967	0.0000000
H	0.0000000	4.2136530	2.1427012
C	0.0000000	1.5236074	2.5266829
C	0.0000000	1.5236074	-2.5266829
H	0.0000000	2.2240418	3.3888377
H	-0.9045789	0.8832700	2.5945640
H	0.9045789	0.8832699	2.5945640
H	0.0000000	2.2240418	-3.3888377
H	0.9045789	0.8832699	-2.5945640
H	-0.9045789	0.8832700	-2.5945640
H	1.0088070	6.2150595	0.0000000
H	-0.5044031	6.2150650	-0.8736506
H	-0.5044031	6.2150650	0.8736506

Coordinates of models from Fig. 1 (PBE1PBE/6-311+G* for Cu and PBE1PBE/6-31G* for C, H optimized)

Monomer

Cu	0.006001	-2.318429	0.000000
C	0.001318	1.682581	1.195888
C	-0.002171	2.399866	-0.000000
C	0.001318	1.682581	-1.195888
C	0.001318	0.284906	-1.214359
C	0.000313	-0.419853	0.000000
C	0.001318	0.284906	1.214359
H	0.005249	2.225305	2.140680
C	-0.036208	3.903568	-0.000000
H	0.005249	2.225305	-2.140680
C	0.008494	-0.436956	-2.537202
C	0.008494	-0.436956	2.537202
H	-0.008297	0.262604	-3.379926
H	0.903412	-1.063491	-2.642023
H	-0.863674	-1.096019	-2.633293
H	-0.008297	0.262604	3.379926
H	-0.863674	-1.096019	2.633293
H	0.903412	-1.063491	2.642023
H	-1.068494	4.277881	-0.000000
H	0.457965	4.315956	0.886128
H	0.457965	4.315956	-0.886128

Dimer

Cu	-0.553017	0.986704	0.468042
C	1.730998	2.772632	1.038348
C	1.945935	3.542468	-0.098666
C	2.024248	2.876226	-1.328606
C	1.821023	1.502376	-1.442048
C	1.560366	0.696551	-0.297093
C	1.578666	1.369059	0.964082
H	1.722578	3.253586	2.016395
C	2.106117	5.035230	-0.020342
H	2.231408	3.457964	-2.226855
C	1.926788	0.855709	-2.798603
C	1.649451	0.577425	2.253014
H	2.798208	0.190927	-2.840211
H	1.047884	0.236879	-3.016013
H	2.027481	1.595806	-3.599972
H	2.701447	0.397988	2.512324
H	1.191189	1.105378	3.097592
H	1.178042	-0.406721	2.149398
H	1.280893	5.552872	-0.525295
H	2.129082	5.380871	1.017916
H	3.033955	5.362665	-0.504238
Cu	0.553017	-0.986704	-0.468042
C	-2.024248	-2.876226	1.328606
C	-1.945935	-3.542468	0.098666
C	-1.730998	-2.772632	-1.038348
C	-1.578666	-1.369059	-0.964082
C	-1.560366	-0.696551	0.297093
C	-1.821023	-1.502376	1.442048
H	-2.231408	-3.457964	2.226855
C	-2.106117	-5.035230	0.020342
H	-1.722578	-3.253586	-2.016395
C	-1.649451	-0.577425	-2.253014
C	-1.926788	-0.855709	2.798603
H	-2.701447	-0.397988	-2.512324
H	-1.178042	0.406721	-2.149398
H	-1.191189	-1.105378	-3.097592
H	-2.798208	-0.190927	2.840211
H	-2.027481	-1.595806	3.599972
H	-1.047884	-0.236879	3.016013
H	-1.280893	-5.552872	0.525295
H	-3.033955	-5.362665	0.504238
H	-2.129082	-5.380871	-1.017916

Dimer planar

Cu	0.007227	1.381366	-0.000000
C	0.037686	5.376428	1.196885
C	0.040285	6.091758	-0.000000
C	0.037686	5.376428	-1.196885
C	0.025854	3.977681	-1.219853
C	0.018871	3.290211	-0.000000
C	0.025854	3.977681	1.219853
H	0.046343	5.918514	2.141789
C	0.019498	7.595595	-0.000000
H	0.046343	5.918514	-2.141789
C	0.025854	3.252255	-2.539640
C	0.025854	3.252255	2.539640
H	0.024853	3.951485	-3.382327
H	0.910014	2.610231	-2.638615
H	-0.857602	2.609101	-2.637396
H	0.024853	3.951485	3.382327
H	-0.857602	2.609101	2.637396
H	0.910014	2.610231	2.638615
H	-1.009879	7.977329	-0.000000
H	0.516971	8.003694	0.885606
H	0.516971	8.003694	-0.885606
Cu	-0.007227	-1.381366	-0.000000
C	-0.037686	-5.376428	1.196885
C	-0.040285	-6.091758	-0.000000
C	-0.037686	-5.376428	-1.196885
C	-0.025854	-3.977681	-1.219853
C	-0.018871	-3.290211	-0.000000
C	-0.025854	-3.977681	1.219853
H	-0.046343	-5.918514	2.141789
C	-0.019498	-7.595595	-0.000000
H	-0.046343	-5.918514	-2.141789
C	-0.025854	-3.252255	-2.539640
C	-0.025854	-3.252255	2.539640
H	-0.024853	-3.951485	-3.382327
H	-0.910014	-2.610231	-2.638615
H	0.857602	-2.609101	-2.637396
H	-0.024853	-3.951485	3.382327
H	0.857602	-2.609101	2.637396
H	-0.910014	-2.610231	2.638615
H	1.009879	-7.977329	-0.000000
H	-0.516971	-8.003694	0.885606
H	-0.516971	-8.003694	-0.885606

Trimer

Cu	0.879327	1.085655	0.000000
C	4.430200	-0.090054	1.196549
C	5.136500	0.033801	0.000000
C	4.430200	-0.090054	-1.196549
C	3.052980	-0.315985	-1.211618
C	2.312014	-0.422351	0.000000
C	3.052980	-0.315985	1.211618
H	4.969258	-0.010510	2.140038
C	6.611777	0.319675	0.000000
H	4.969258	-0.010510	-2.140038
C	2.376693	-0.506238	-2.546833
C	2.376693	-0.506238	2.546833
H	2.974778	-0.094959	-3.367371
H	2.222230	-1.573170	-2.755113
H	1.391741	-0.027458	-2.571853
H	2.974778	-0.094959	3.367371
H	1.391741	-0.027458	2.571853
H	2.222230	-1.573170	2.755113
H	6.801222	1.401165	0.000000
H	7.101048	-0.095836	0.887069
H	7.101048	-0.095836	-0.887069
Cu	0.500541	-1.304347	0.000000
C	-2.293089	-3.791639	1.196549
C	-2.538978	-4.465240	0.000000
C	-2.293089	-3.791639	-1.196549
C	-1.800141	-2.485966	-1.211618
C	-1.521774	-1.791087	0.000000
C	-1.800141	-2.485966	1.211618
H	-2.493731	-4.298248	2.140038
C	-3.029042	-5.885804	0.000000
H	-2.493731	-4.298248	-2.140038
C	-1.626762	-1.805157	-2.546833
C	-1.626762	-1.805157	2.546833
H	-1.569626	-2.528754	-3.367371
H	-2.473520	-1.137922	-2.755113
H	-0.719649	-1.191554	-2.571853
H	-1.569626	-2.528754	3.367371
H	-0.719649	-1.191554	2.571853
H	-2.473520	-1.137922	2.755113
H	-2.187166	-6.590614	0.000000
H	-3.633521	-6.101770	0.887069
H	-3.633521	-6.101770	-0.887069
Cu	-1.379868	0.218692	0.000000
C	-2.137111	3.881693	1.196549
C	-2.597522	4.431439	0.000000
C	-2.137111	3.881693	-1.196549
C	-1.252839	2.801951	-1.211618
C	-0.790240	2.213438	0.000000
C	-1.252839	2.801951	1.211618

H	-2.475527	4.308758	2.140038
C	-3.582735	5.566130	0.000000
H	-2.475527	4.308758	-2.140038
C	-0.749931	2.311395	-2.546833
C	-0.749931	2.311395	2.546833
H	-1.405152	2.623713	-3.367371
H	0.251290	2.711092	-2.755113
H	-0.672091	1.219012	-2.571853
H	-1.405152	2.623713	3.367371
H	-0.672091	1.219012	2.571853
H	0.251290	2.711092	2.755113
H	-4.614056	5.189449	0.000000
H	-3.467527	6.197606	0.887069
H	-3.467527	6.197606	-0.887069

Tetramer

Cu	-1.173899	-1.274226	-0.010859
C	0.081379	-5.111769	-0.536813
C	0.006478	-5.606762	0.769394
C	0.000249	-4.693286	1.818851
C	0.059389	-3.314503	1.590690
C	0.122052	-2.798591	0.269325
C	0.141167	-3.745973	-0.796348
H	0.091902	-5.815137	-1.369005
C	-0.081379	-7.085773	1.021541
H	-0.053935	-5.062661	2.842648
C	0.081634	-2.404589	2.794656
C	0.265389	-3.289406	-2.229186
H	-0.680456	-2.689405	3.530109
H	1.052687	-2.452318	3.305386
H	-0.092963	-1.360950	2.510646
H	-0.064364	-4.065189	-2.928974
H	-0.326565	-2.386764	-2.417414
H	1.307754	-3.047132	-2.475484
H	-1.049496	-7.484965	0.693449
H	0.693791	-7.630976	0.470718
H	0.030970	-7.318886	2.084842
Cu	1.274226	-1.173899	0.010859
C	4.693286	0.000249	-1.818851
C	5.606762	0.006478	-0.769394
C	5.111769	0.081379	0.536813
C	3.745973	0.141167	0.796348
C	2.798591	0.122052	-0.269325
C	3.314503	0.059389	-1.590690
H	5.062661	-0.053935	-2.842648
C	7.085773	-0.081379	-1.021541
H	5.815137	0.091902	1.369005
C	3.289406	0.265389	2.229186
C	2.404589	0.081634	-2.794656
H	4.065189	-0.064364	2.928974

H	3.047132	1.307754	2.475484
H	2.386764	-0.326565	2.417414
H	2.689405	-0.680456	-3.530109
H	1.360950	-0.092963	-2.510646
H	2.452318	1.052687	-3.305386
H	7.484965	-1.049496	-0.693449
H	7.318886	0.030970	-2.084842
H	7.630976	0.693791	-0.470718
Cu	1.173899	1.274226	-0.010859
C	-0.081379	5.111769	-0.536813
C	-0.006478	5.606762	0.769394
C	-0.000249	4.693286	1.818851
C	-0.059389	3.314503	1.590690
C	-0.122052	2.798591	0.269325
C	-0.141167	3.745973	-0.796348
H	-0.091902	5.815137	-1.369005
C	0.081379	7.085773	1.021541
H	0.053935	5.062661	2.842648
C	-0.081634	2.404589	2.794656
C	-0.265389	3.289406	-2.229186
H	0.680456	2.689405	3.530109
H	-1.052687	2.452318	3.305386
H	0.092963	1.360950	2.510646
H	0.064364	4.065189	-2.928974
H	0.326565	2.386764	-2.417414
H	-1.307754	3.047132	-2.475484
H	1.049496	7.484965	0.693449
H	-0.693791	7.630976	0.470718
H	-0.030970	7.318886	2.084842
Cu	-1.274226	1.173899	0.010859
C	-4.693286	-0.000249	-1.818851
C	-5.606762	-0.006478	-0.769394
C	-5.111769	-0.081379	0.536813
C	-3.745973	-0.141167	0.796348
C	-2.798591	-0.122052	-0.269325
C	-3.314503	-0.059389	-1.590690
H	-5.062661	0.053935	-2.842648
C	-7.085773	0.081379	-1.021541
H	-5.815137	-0.091902	1.369005
C	-3.289406	-0.265389	2.229186
C	-2.404589	-0.081634	-2.794656
H	-4.065189	0.064364	2.928974
H	-3.047132	-1.307754	2.475484
H	-2.386764	0.326565	2.417414
H	-2.689405	0.680456	-3.530109
H	-1.360950	0.092963	-2.510646
H	-2.452318	-1.052687	-3.305386
H	-7.484965	1.049496	-0.693449
H	-7.318886	-0.030970	-2.084842
H	-7.630976	-0.693791	-0.470718

Pentamer

Cu	-0.217578	-2.111021	0.000362
C	2.151187	-3.828895	-2.904930
C	2.758545	-4.868695	-2.207734
C	2.729232	-4.832835	-0.809284
C	2.117686	-3.790809	-0.119148
C	1.498402	-2.717082	-0.823890
C	1.525051	-2.767937	-2.241637
H	2.166146	-3.841370	-3.994420
C	3.442371	-5.997089	-2.927595
H	3.201067	-5.639719	-0.249351
C	2.102431	-3.826512	1.389533
C	0.862823	-1.701184	-3.078328
H	2.781641	-4.592081	1.779936
H	2.399968	-2.860585	1.814993
H	1.096665	-4.050208	1.769519
H	1.459330	-1.456431	-3.965122
H	-0.122398	-2.031500	-3.434446
H	0.710720	-0.781700	-2.502269
H	3.062572	-6.970089	-2.594218
H	3.295110	-5.930060	-4.009886
H	4.522671	-5.991616	-2.736012
Cu	1.947942	-0.899421	-0.107297
C	5.464667	1.211263	-0.122156
C	5.836550	1.228999	1.226709
C	4.874915	0.901811	2.176810
C	3.564688	0.572024	1.812639
C	3.171908	0.569498	0.449342
C	4.170819	0.888402	-0.518119
H	6.208898	1.456484	-0.879267
C	7.235356	1.604682	1.628026
H	5.149568	0.903353	3.231419
C	2.603064	0.197550	2.912946
C	3.853052	0.847133	-1.992613
H	2.627510	0.923347	3.734922
H	1.575417	0.135964	2.541019
H	2.858444	-0.779864	3.343847
H	4.637092	1.330497	-2.585682
H	3.761285	-0.188424	-2.346489
H	2.903181	1.346776	-2.214380
H	7.979924	1.034272	1.060647
H	7.429324	2.667346	1.434883
H	7.409469	1.423293	2.693114
Cu	1.433238	1.532432	0.137026
C	0.527382	5.332434	-1.192868
C	0.451468	6.049929	-0.001728
C	0.332146	5.334534	1.192672
C	0.283363	3.942540	1.208777
C	0.344384	3.199265	-0.005506
C	0.474477	3.936240	-1.212897

H	0.624866	5.875203	-2.132909
C	0.470721	7.552851	0.002704
H	0.280299	5.881117	2.134041
C	0.217079	3.244112	2.544425
C	0.559438	3.249109	-2.553485
H	-0.324204	3.842264	3.286469
H	-0.274955	2.268376	2.465108
H	1.225266	3.067269	2.942224
H	-0.230402	3.597185	-3.230981
H	1.518532	3.455830	-3.045710
H	0.461360	2.162743	-2.451917
H	1.060592	7.943448	0.839142
H	0.890941	7.951249	-0.926120
H	-0.544185	7.958446	0.105586
Cu	-1.063845	1.786101	-0.164890
C	-4.650447	1.598018	-2.194737
C	-5.550283	2.104621	-1.262347
C	-5.147569	2.169456	0.075679
C	-3.887249	1.740419	0.480446
C	-2.960417	1.212216	-0.465817
C	-3.375825	1.158467	-1.821780
H	-4.948378	1.539355	-3.241315
C	-6.924354	2.558230	-1.668900
H	-5.840893	2.563183	0.818397
C	-3.511641	1.873603	1.935586
C	-2.458825	0.652842	-2.907625
H	-4.396704	2.003403	2.567908
H	-2.966322	0.992191	2.293196
H	-2.861810	2.744056	2.095937
H	-2.987113	-0.007192	-3.606215
H	-2.050277	1.483994	-3.497496
H	-1.612390	0.098911	-2.488449
H	-7.124173	3.580123	-1.325523
H	-7.048260	2.537151	-2.755992
H	-7.698663	1.915552	-1.231584
Cu	-2.072679	-0.470973	0.144593
C	-3.616775	-4.194621	0.840999
C	-3.571340	-4.287923	2.237081
C	-2.747725	-3.405793	2.928275
C	-1.976640	-2.446308	2.261806
C	-2.017157	-2.342220	0.848108
C	-2.866574	-3.250020	0.148704
H	-4.259496	-4.877258	0.285962
C	-4.399035	-5.313684	2.959421
H	-2.702567	-3.463773	4.015511
C	-1.093327	-1.548484	3.092566
C	-2.975820	-3.190089	-1.355326
H	-0.213515	-2.089493	3.465799
H	-0.730586	-0.697541	2.505795
H	-1.626671	-1.159912	3.968694
H	-3.600982	-4.001001	-1.744302

H	-3.418308	-2.240540	-1.683123
H	-1.990085	-3.268117	-1.830858
H	-5.469895	-5.155425	2.782075
H	-4.163684	-6.328014	2.615178
H	-4.229399	-5.277277	4.039881

Hexamer

Cu	2.167231	1.288642	0.008825
C	4.810006	0.040923	2.872217
C	6.012552	0.041949	2.172263
C	5.963192	0.046820	0.773829
C	4.753755	0.047085	0.085582
C	3.516216	0.045331	0.795415
C	3.576003	0.046467	2.212278
H	4.830565	0.036533	3.961634
C	7.333889	0.032686	2.888683
H	6.896308	0.045523	0.211336
C	4.772597	0.038661	-1.423524
C	2.322277	0.085093	3.048971
H	5.795558	0.098175	-1.810411
H	4.318407	-0.878378	-1.821077
H	4.208220	0.885539	-1.834256
H	2.422237	-0.526904	3.953220
H	2.095166	1.108839	3.375891
H	1.457290	-0.275849	2.483106
H	7.943859	0.899807	2.607936
H	7.201227	0.052522	3.974735
H	7.915213	-0.862937	2.637503
Cu	2.195976	-1.231499	0.007421
C	3.032795	-5.134734	-0.762435
C	3.053477	-5.175714	-2.161289
C	2.446219	-4.136040	-2.858109
C	1.827789	-3.070019	-2.194683
C	1.796988	-3.022886	-0.777895
C	2.422920	-4.092674	-0.071087
H	3.503245	-5.942367	-0.202514
C	3.709479	-6.320668	-2.881009
H	2.453380	-4.152243	-3.947636
C	1.225175	-1.967985	-3.028575
C	2.426213	-4.116511	1.437869
H	0.688371	-2.367392	-3.897463
H	0.525840	-1.365622	-2.439719
H	2.000392	-1.291109	-3.412366
H	2.988157	-4.974393	1.822463
H	2.879264	-3.205943	1.850307
H	1.405149	-4.180998	1.835961
H	4.754685	-6.438583	-2.571027
H	3.201715	-7.268516	-2.663645
H	3.694132	-6.174112	-3.965359

Cu	0.030175	-2.520531	0.009121
C	-2.379472	-4.185939	2.862119
C	-2.975099	-5.230249	2.161766
C	-2.941551	-5.191912	0.763341
C	-2.336910	-4.144228	0.075545
C	-1.724970	-3.068783	0.785766
C	-1.758566	-3.119204	2.202566
H	-2.396610	-4.200170	3.951521
C	-3.646009	-6.368956	2.877639
H	-3.405011	-6.001575	0.200533
C	-2.346924	-4.159871	-1.433567
C	-1.104631	-2.049573	3.040189
H	-2.814899	-5.071533	-1.820268
H	-2.902989	-3.303056	-1.835889
H	-1.328775	-4.106773	-1.839799
H	-1.695627	-1.820922	3.935030
H	-0.110429	-2.366774	3.382953
H	-0.973660	-1.124662	2.469160
H	-3.223162	-7.333940	2.573507
H	-3.536674	-6.280330	3.962863
H	-4.718690	-6.403053	2.650394
Cu	-2.167231	-1.288642	-0.008825
C	-5.963192	-0.046820	-0.773829
C	-6.012552	-0.041949	-2.172263
C	-4.810006	-0.040923	-2.872217
C	-3.576003	-0.046467	-2.212278
C	-3.516216	-0.045331	-0.795415
C	-4.753755	-0.047085	-0.085582
H	-6.896308	-0.045523	-0.211336
C	-7.333889	-0.032686	-2.888683
H	-4.830565	-0.036533	-3.961634
C	-2.322277	-0.085093	-3.048971
C	-4.772597	-0.038661	1.423524
H	-2.422237	0.526904	-3.953220
H	-1.457290	0.275849	-2.483106
H	-2.095166	-1.108839	-3.375891
H	-5.795558	-0.098175	1.810411
H	-4.208220	-0.885539	1.834256
H	-4.318407	0.878378	1.821077
H	-7.943859	-0.899807	-2.607936
H	-7.915213	0.862937	-2.637503
H	-7.201227	-0.052522	-3.974735
Cu	-2.195976	1.231499	-0.007421
C	-2.446219	4.136040	2.858109
C	-3.053477	5.175714	2.161289
C	-3.032795	5.134734	0.762435
C	-2.422920	4.092674	0.071087
C	-1.796988	3.022886	0.777895
C	-1.827789	3.070019	2.194683
H	-2.453380	4.152243	3.947636
C	-3.709479	6.320668	2.881009

H	-3.503245	5.942367	0.202514
C	-2.426213	4.116511	-1.437869
C	-1.225175	1.967985	3.028575
H	-2.988157	4.974393	-1.822463
H	-1.405149	4.180998	-1.835961
H	-2.879264	3.205943	-1.850307
H	-0.688371	2.367392	3.897463
H	-2.000392	1.291109	3.412366
H	-0.525840	1.365622	2.439719
H	-4.754685	6.438583	2.571027
H	-3.694132	6.174112	3.965359
H	-3.201715	7.268516	2.663645
Cu	-0.030175	2.520531	-0.009121
C	2.941551	5.191912	-0.763341
C	2.975099	5.230249	-2.161766
C	2.379472	4.185939	-2.862119
C	1.758566	3.119204	-2.202566
C	1.724970	3.068783	-0.785766
C	2.336910	4.144228	-0.075545
H	3.405011	6.001575	-0.200533
C	3.646009	6.368956	-2.877639
H	2.396610	4.200170	-3.951521
C	1.104631	2.049573	-3.040189
C	2.346924	4.159871	1.433567
H	1.695627	1.820922	-3.935030
H	0.973660	1.124662	-2.469160
H	0.110429	2.366774	-3.382953
H	2.814899	5.071533	1.820268
H	1.328775	4.106773	1.839799
H	2.902989	3.303056	1.835889
H	3.223162	7.333940	-2.573507
H	4.718690	6.403053	-2.650394
H	3.536674	6.280330	-3.962863

Heptamer

Cu	-1.828291	-2.189371	-0.345733
C	-4.937172	-2.842027	2.227121
C	-5.936472	-3.262761	1.354320
C	-5.788049	-2.975902	-0.006660
C	-4.680913	-2.285579	-0.492176
C	-3.652080	-1.852171	0.395816
C	-3.808038	-2.152126	1.773044
H	-5.036436	-3.057873	3.290469
C	-7.151166	-3.994703	1.852502
H	-6.562461	-3.296520	-0.702807
C	-4.596496	-1.990369	-1.969686
C	-2.751860	-1.772901	2.779178
H	-5.416753	-2.465204	-2.518703
H	-4.648833	-0.910387	-2.160627

H	-3.652563	-2.351345	-2.397294
H	-2.044600	-2.597941	2.937094
H	-2.170573	-0.909181	2.438861
H	-3.192042	-1.531369	3.753733
H	-7.300506	-4.934859	1.308518
H	-7.068121	-4.230605	2.917882
H	-8.059496	-3.395380	1.712706
Cu	-3.055080	0.009592	0.000066
C	-5.768674	3.012708	0.004864
C	-5.914033	3.301033	-1.356122
C	-4.916739	2.874091	-2.228222
C	-3.792575	2.176607	-1.773442
C	-3.639774	1.875112	-0.396185
C	-4.666533	2.314898	0.491074
H	-6.541551	3.338186	0.700461
C	-7.123677	4.040698	-1.855183
H	-5.013640	3.091018	-3.291568
C	-2.738079	1.790723	-2.778812
C	-4.585519	2.018417	1.968519
H	-3.178987	1.553025	-3.753981
H	-2.024865	2.610873	-2.935420
H	-2.163230	0.922669	-2.438592
H	-5.402809	2.498899	2.517051
H	-4.645835	0.938749	2.158902
H	-3.639389	2.372359	2.397141
H	-8.035115	3.444682	-1.721463
H	-7.270874	4.978927	-1.307342
H	-7.035835	4.281107	-2.919173
Cu	-1.814377	2.200487	0.346810
C	-0.408726	3.954808	3.767401
C	-0.716731	5.291441	3.535512
C	-0.979669	5.690727	2.220010
C	-0.938023	4.790041	1.160149
C	-0.621424	3.417068	1.390031
C	-0.360583	3.021273	2.725375
H	-0.198088	3.627434	4.785154
C	-0.763422	6.289159	4.658817
H	-1.219266	6.735027	2.022038
C	-1.234959	5.287988	-0.233404
C	-0.044494	1.588559	3.067219
H	-1.399226	6.370860	-0.242855
H	-0.409095	5.067069	-0.921462
H	-2.134575	4.810159	-0.642850
H	-0.915283	1.082503	3.505614
H	0.249693	1.025523	2.175049
H	0.768353	1.519627	3.800289
H	-1.752517	6.757234	4.733068
H	-0.541117	5.819317	5.621803
H	-0.037753	7.096769	4.503105
Cu	0.660465	2.699808	0.037217
C	3.512531	5.003573	-1.640322

C	3.175879	4.981458	-2.997283
C	2.355899	3.953050	-3.453057
C	1.869103	2.964902	-2.591186
C	2.194790	2.985600	-1.210940
C	3.045161	4.036534	-0.754083
H	4.155895	5.799950	-1.267390
C	3.676914	6.050996	-3.927017
H	2.084140	3.919388	-4.507715
C	1.020299	1.866100	-3.175209
C	3.476474	4.109493	0.690121
H	0.393956	2.231950	-3.996982
H	1.643802	1.057566	-3.579180
H	0.368244	1.424060	-2.415504
H	3.953116	5.069533	0.915965
H	2.626972	3.985886	1.373011
H	4.199883	3.316940	0.923446
H	3.224743	7.022905	-3.692398
H	4.763060	6.174263	-3.844168
H	3.441596	5.817887	-4.970063
Cu	2.669469	1.219204	-0.401439
C	6.315860	0.287216	1.154348
C	7.030727	-0.025656	-0.002327
C	6.312339	-0.338073	-1.157089
C	4.917249	-0.328655	-1.176704
C	4.180718	-0.014247	-0.001142
C	4.920637	0.292058	1.174288
H	6.862148	0.523071	2.067227
C	8.533622	-0.000632	-0.011921
H	6.855990	-0.587439	-2.067988
C	4.215104	-0.609908	-2.482053
C	4.221844	0.572314	2.481675
H	4.072979	0.317573	-3.053708
H	4.794267	-1.294716	-3.112098
H	3.224211	-1.048631	-2.318569
H	4.806674	1.249806	3.114380
H	3.234316	1.019561	2.320761
H	4.072797	-0.356652	3.049156
H	8.908045	0.991734	-0.295544
H	8.945253	-0.233756	0.975532
H	8.941746	-0.719105	-0.730443
Cu	2.661371	-1.237534	0.401149
C	2.330120	-3.966089	3.455867
C	3.144894	-4.999277	3.001544
C	3.482029	-5.024608	1.644780
C	3.020257	-4.055981	0.757328
C	2.175210	-3.000107	1.212676
C	1.848984	-2.976273	2.592753
H	2.057971	-3.929965	4.510344
C	3.640146	-6.070293	3.932671
H	4.121386	-5.824730	1.272980
C	3.452596	-4.132442	-0.686377

C	1.005622	-1.872496	3.175249
H	3.917984	-5.097868	-0.912732
H	2.605637	-3.997685	-1.370282
H	4.185974	-3.348460	-0.917586
H	1.633164	-1.067623	3.580268
H	0.357449	-1.426725	2.414404
H	0.375748	-2.234623	3.995973
H	4.726219	-6.196432	3.853108
H	3.402328	-5.836631	4.975024
H	3.186122	-7.040958	3.696538
Cu	0.643186	-2.706035	-0.036227
C	-1.019614	-5.684041	-2.220726
C	-0.754536	-5.285857	-3.536131
C	-0.436675	-3.951418	-3.767335
C	-0.380707	-3.018986	-2.724707
C	-0.643551	-3.413733	-1.389442
C	-0.970421	-4.784415	-1.160283
H	-1.267056	-6.726616	-2.023310
C	-0.808859	-6.282564	-4.659992
H	-0.224367	-3.624940	-4.785031
C	-0.054009	-1.588494	-3.065903
C	-1.270839	-5.280939	0.233029
H	0.760438	-1.525250	-3.797717
H	-0.920516	-1.076301	-3.505651
H	0.242806	-1.027622	-2.173236
H	-1.439467	-6.363141	0.242482
H	-0.444898	-5.063174	0.921973
H	-2.169033	-4.799473	0.641377
H	-0.083816	-7.091591	-4.508597
H	-1.799121	-6.748824	-4.729583
H	-0.590441	-5.812380	-5.623700

Coordinates of models optimized with MN15/6-311+G* for Cu and MN15/6-31G* for C, H.

Monomer

Cu	0.006003	-2.322446	0.000000
C	0.001209	1.688346	1.199094
C	-0.003214	2.405491	-0.000000
C	0.001209	1.688346	-1.199094
C	0.001209	0.288939	-1.215482
C	-0.000026	-0.415875	0.000000
C	0.001209	0.288939	1.215482
H	0.006392	2.232510	2.144891
C	-0.036747	3.913623	-0.000000
H	0.006392	2.232510	-2.144891
C	0.009039	-0.444798	-2.536870
C	0.009039	-0.444798	2.536870
H	-0.006052	0.249246	-3.385268
H	0.904447	-1.073773	-2.631482
H	-0.864984	-1.104165	-2.624272
H	-0.006052	0.249246	3.385268
H	-0.864984	-1.104165	2.624272
H	0.904447	-1.073773	2.631482
H	-1.068707	4.288528	-0.000000
H	0.458729	4.322746	0.887613
H	0.458729	4.322746	-0.887613

Dimer

Cu	-0.317586	1.651440	-0.013770
C	1.628164	2.006534	1.226228
C	1.615491	2.736832	0.016985
C	1.671061	1.995974	-1.184614
C	1.690058	0.584410	-1.180223
C	1.593734	-0.153183	0.029298
C	1.647092	0.595028	1.234931
H	1.620953	2.553727	2.169868
C	1.652201	4.245644	0.010983
H	1.697491	2.534849	-2.132692
C	1.751810	-0.143839	-2.502192
C	1.661569	-0.121501	2.564650
H	2.730547	-0.624427	-2.623405
H	0.998152	-0.941773	-2.536558
H	1.592390	0.529401	-3.353323
H	2.634908	-0.601765	2.724477
H	1.473046	0.559425	3.403636
H	0.906264	-0.918489	2.579624
H	1.141844	4.657631	0.888153
H	2.687764	4.608871	0.027356
H	1.172699	4.649830	-0.886984
Cu	0.317586	-1.651440	0.013770
C	-1.671061	-1.995974	1.184614

C	-1.615491	-2.736832	-0.016985
C	-1.628164	-2.006534	-1.226228
C	-1.647092	-0.595028	-1.234931
C	-1.593734	0.153183	-0.029298
C	-1.690058	-0.584410	1.180223
H	-1.697491	-2.534849	2.132692
C	-1.652201	-4.245644	-0.010983
H	-1.620953	-2.553727	-2.169868
C	-1.661569	0.121501	-2.564650
C	-1.751810	0.143839	2.502192
H	-2.634908	0.601765	-2.724477
H	-0.906264	0.918489	-2.579624
H	-1.473046	-0.559425	-3.403636
H	-2.730547	0.624427	2.623405
H	-1.592390	-0.529401	3.353323
H	-0.998152	0.941773	2.536558
H	-1.141844	-4.657631	-0.888153
H	-1.172699	-4.649830	0.886984
H	-2.687764	-4.608871	-0.027356

Trimer

Cu	0.957396	1.031269	0.000000
C	4.353243	-0.097551	1.200778
C	5.049815	0.068256	0.000000
C	4.353243	-0.097551	-1.200778
C	2.990067	-0.409228	-1.213540
C	2.265886	-0.576635	0.000000
C	2.990067	-0.409228	1.213540
H	4.887594	0.015020	2.145181
C	6.510509	0.440702	0.000000
H	4.887594	0.015020	-2.145181
C	2.292217	-0.617664	-2.540831
C	2.292217	-0.617664	2.540831
H	2.953370	-0.396196	-3.386594
H	1.949148	-1.656675	-2.639250
H	1.404483	0.024660	-2.626433
H	2.953370	-0.396196	3.386594
H	1.404483	0.024660	2.626433
H	1.949148	-1.656675	2.639250
H	6.636861	1.531184	0.000000
H	7.020301	0.052251	0.888422
H	7.020301	0.052251	-0.888422
Cu	0.414407	-1.344764	0.000000
C	-2.261103	-3.721244	1.200778
C	-2.465796	-4.407396	0.000000
C	-2.261103	-3.721244	-1.200778
C	-1.849435	-2.384860	-1.213540
C	-1.632323	-1.673997	0.000000
C	-1.849435	-2.384860	1.213540
H	-2.430789	-4.240290	2.145181

C	-2.873595	-5.858617	0.000000
H	-2.430789	-4.240290	-2.145181
C	-1.681021	-1.676287	-2.540831
C	-1.681021	-1.676287	2.540831
H	-1.819801	-2.359595	-3.386594
H	-2.409297	-0.859674	-2.639250
H	-0.680885	-1.228648	-2.626433
H	-1.819801	-2.359595	3.386594
H	-0.680885	-1.228648	2.626433
H	-2.409297	-0.859674	2.639250
H	-1.992386	-6.513282	0.000000
H	-3.464900	-6.105885	0.888422
H	-3.464900	-6.105885	-0.888422
Cu	-1.371803	0.313495	0.000000
C	-2.092140	3.818795	1.200778
C	-2.584019	4.339140	0.000000
C	-2.092140	3.818795	-1.200778
C	-1.140632	2.794088	-1.213540
C	-0.633563	2.250632	0.000000
C	-1.140632	2.794088	1.213540
H	-2.456804	4.225270	2.145181
C	-3.636913	5.417915	0.000000
H	-2.456804	4.225270	-2.145181
C	-0.611196	2.293950	-2.540831
C	-0.611196	2.293950	2.540831
H	-1.133569	2.755792	-3.386594
H	0.460148	2.516350	-2.639250
H	-0.723598	1.203988	-2.626433
H	-1.133569	2.755792	3.386594
H	-0.723598	1.203988	2.626433
H	0.460148	2.516350	2.639250
H	-4.644474	4.982098	0.000000
H	-3.555402	6.053634	0.888422
H	-3.555402	6.053634	-0.888422

Tetramer

Cu	-1.111360	1.316250	-0.035282
C	-0.068403	4.564233	1.993799
C	-0.031027	5.529082	0.987952
C	0.153831	5.109196	-0.337931
C	0.284703	3.759574	-0.656839
C	0.237468	2.765469	0.362081
C	0.068403	3.200121	1.700944
H	-0.205235	4.880523	3.029043
C	-0.199971	6.992955	1.306377
H	0.191013	5.857066	-1.131485
C	0.508722	3.344378	-2.095248
C	0.080437	2.207065	2.843874
H	0.361707	4.181536	-2.787439
H	1.530938	2.963661	-2.236847

H	-0.176129	2.533472	-2.383550
H	-0.624553	2.488238	3.636201
H	-0.179166	1.198362	2.491342
H	1.078893	2.148948	3.300000
H	-1.164793	7.366493	0.940627
H	-0.159340	7.172144	2.385877
H	0.581842	7.595639	0.829363
Cu	1.316250	1.111360	0.035282
C	5.109196	-0.153831	0.337931
C	5.529082	0.031027	-0.987952
C	4.564233	0.068403	-1.993799
C	3.200121	-0.068403	-1.700944
C	2.765469	-0.237468	-0.362081
C	3.759574	-0.284703	0.656839
H	5.857066	-0.191013	1.131485
C	6.992955	0.199971	-1.306377
H	4.880523	0.205235	-3.029043
C	2.207065	-0.080437	-2.843874
C	3.344378	-0.508722	2.095248
H	2.488238	0.624553	-3.636201
H	2.148948	-1.078893	-3.300000
H	1.198362	0.179166	-2.491342
H	4.181536	-0.361707	2.787439
H	2.533472	0.176129	2.383550
H	2.963661	-1.530938	2.236847
H	7.366493	1.164793	-0.940627
H	7.595639	-0.581842	-0.829363
H	7.172144	0.159340	-2.385877
Cu	1.111360	-1.316250	-0.035282
C	0.068403	-4.564233	1.993799
C	0.031027	-5.529082	0.987952
C	-0.153831	-5.109196	-0.337931
C	-0.284703	-3.759574	-0.656839
C	-0.237468	-2.765469	0.362081
C	-0.068403	-3.200121	1.700944
H	0.205235	-4.880523	3.029043
C	0.199971	-6.992955	1.306377
H	-0.191013	-5.857066	-1.131485
C	-0.508722	-3.344378	-2.095248
C	-0.080437	-2.207065	2.843874
H	-0.361707	-4.181536	-2.787439
H	-1.530938	-2.963661	-2.236847
H	0.176129	-2.533472	-2.383550
H	0.624553	-2.488238	3.636201
H	0.179166	-1.198362	2.491342
H	-1.078893	-2.148948	3.300000
H	1.164793	-7.366493	0.940627
H	0.159340	-7.172144	2.385877
H	-0.581842	-7.595639	0.829363
Cu	-1.316250	-1.111360	0.035282
C	-5.109196	0.153831	0.337931

C	-5.529082	-0.031027	-0.987952
C	-4.564233	-0.068403	-1.993799
C	-3.200121	0.068403	-1.700944
C	-2.765469	0.237468	-0.362081
C	-3.759574	0.284703	0.656839
H	-5.857066	0.191013	1.131485
C	-6.992955	-0.199971	-1.306377
H	-4.880523	-0.205235	-3.029043
C	-2.207065	0.080437	-2.843874
C	-3.344378	0.508722	2.095248
H	-2.488238	-0.624553	-3.636201
H	-2.148948	1.078893	-3.300000
H	-1.198362	-0.179166	-2.491342
H	-4.181536	0.361707	2.787439
H	-2.533472	-0.176129	2.383550
H	-2.963661	1.530938	2.236847
H	-7.366493	-1.164793	-0.940627
H	-7.595639	0.581842	-0.829363
H	-7.172144	-0.159340	-2.385877

Pentamer

Cu	0.037085	-2.138675	-0.046835
C	3.128916	-4.536614	1.165048
C	2.906160	-4.579147	2.547257
C	2.099912	-3.595006	3.124182
C	1.512343	-2.589467	2.348087
C	1.733298	-2.540890	0.947823
C	2.560479	-3.541284	0.368911
H	3.765695	-5.294232	0.705827
C	3.510017	-5.676325	3.386771
H	1.925715	-3.613929	4.201008
C	0.603801	-1.576439	3.010212
C	2.857388	-3.506829	-1.114819
H	0.820104	-1.472356	4.080239
H	0.699445	-0.589321	2.532571
H	-0.451300	-1.873581	2.911028
H	3.324068	-4.438046	-1.456325
H	1.936311	-3.349375	-1.695568
H	3.540797	-2.679232	-1.357744
H	2.927572	-6.602723	3.300546
H	4.532899	-5.905033	3.066883
H	3.534466	-5.398573	4.445889
Cu	2.024831	-0.727948	0.158989
C	4.770980	0.980731	-2.271051
C	5.693881	1.511999	-1.362402
C	5.286899	1.713559	-0.041183
C	3.994353	1.385249	0.380288
C	3.057782	0.833782	-0.532446
C	3.473882	0.643771	-1.878277
H	5.074434	0.827092	-3.307678

C	7.101124	1.834745	-1.796553
H	5.993341	2.136506	0.674538
C	3.587350	1.668190	1.810532
C	2.513328	0.071013	-2.897794
H	4.459392	1.780477	2.465266
H	3.001529	2.597978	1.871036
H	2.957560	0.859329	2.209654
H	2.973834	0.003581	-3.890449
H	2.181903	-0.936198	-2.606948
H	1.608142	0.687620	-2.980460
H	7.740560	0.943644	-1.750066
H	7.125091	2.199479	-2.829491
H	7.551621	2.596584	-1.151273
Cu	1.212842	1.558366	-0.197718
C	0.154589	5.284482	-1.156105
C	0.284245	5.974739	0.049558
C	0.270334	5.241975	1.243840
C	0.129704	3.854408	1.242197
C	0.006954	3.142908	0.015222
C	0.026257	3.890390	-1.189470
H	0.162127	5.844662	-2.092485
C	0.454758	7.472603	0.073443
H	0.361381	5.771678	2.193502
C	0.045047	3.117771	2.561502
C	-0.038187	3.200680	-2.535288
H	0.547627	3.667852	3.366326
H	-1.004142	2.976393	2.860438
H	0.499052	2.118701	2.486864
H	0.959716	3.144212	-2.993744
H	-0.412319	2.172130	-2.433064
H	-0.689943	3.737983	-3.235495
H	1.494859	7.746263	0.292144
H	0.189874	7.917892	-0.891324
H	-0.171009	7.931975	0.847256
Cu	-1.253234	1.604668	0.187382
C	-5.258093	1.789709	-0.054936
C	-5.618625	1.889471	1.294938
C	-4.715153	1.439466	2.259038
C	-3.468221	0.912172	1.902036
C	-3.083641	0.835280	0.539270
C	-4.020152	1.274540	-0.439319
H	-5.961105	2.124507	-0.819100
C	-6.943978	2.488101	1.692186
H	-4.990112	1.497491	3.313313
C	-2.563105	0.376675	2.988715
C	-3.688325	1.155144	-1.911655
H	-2.724993	0.891468	3.943765
H	-2.748045	-0.694524	3.157433
H	-1.507498	0.480336	2.708396
H	-4.435859	1.659070	-2.535240
H	-2.704984	1.594209	-2.134173

H	-3.646168	0.099416	-2.218712
H	-6.902965	3.584689	1.665966
H	-7.741983	2.176465	1.008772
H	-7.225597	2.191988	2.708334
Cu	-1.990835	-0.726747	-0.043011
C	-2.556595	-3.152303	-3.147817
C	-3.285176	-4.197527	-2.582288
C	-3.219522	-4.388337	-1.192964
C	-2.448976	-3.556005	-0.385136
C	-1.698843	-2.488787	-0.956678
C	-1.762857	-2.305648	-2.359661
H	-2.605227	-2.992678	-4.226126
C	-4.127565	-5.111399	-3.435753
H	-3.792061	-5.199526	-0.740666
C	-2.405934	-3.779020	1.111798
C	-0.962892	-1.212754	-3.036070
H	-3.065273	-4.599127	1.418632
H	-1.385345	-4.018006	1.446477
H	-2.717076	-2.870972	1.650117
H	-1.546982	-0.703850	-3.814027
H	-0.628778	-0.461723	-2.303458
H	-0.062816	-1.621972	-3.516326
H	-5.158532	-5.160997	-3.065476
H	-4.154954	-4.769952	-4.475797
H	-3.731677	-6.134634	-3.427560

Hexamer

Cu	0.001518	-2.652483	-0.000294
C	2.522929	-3.119648	-3.271367
C	3.561483	-3.942967	-2.823546
C	3.664159	-4.207290	-1.452685
C	2.755922	-3.668709	-0.537038
C	1.706385	-2.822249	-0.985014
C	1.604969	-2.561018	-2.376256
H	2.434996	-2.905429	-4.337489
C	4.534325	-4.555892	-3.798841
H	4.473274	-4.845403	-1.093991
C	2.924755	-3.953810	0.941120
C	0.481129	-1.690357	-2.891704
H	3.533283	-4.850344	1.109506
H	3.420056	-3.114498	1.454433
H	1.948097	-4.102384	1.422221
H	0.709142	-1.256519	-3.874148
H	-0.457107	-2.260357	-2.984357
H	0.284534	-0.869733	-2.187303
H	4.200550	-5.554107	-4.110739
H	4.629839	-3.944682	-4.702849
H	5.528083	-4.669244	-3.351659
Cu	1.959827	-1.151348	0.144417
C	5.660336	-1.182613	1.081931

C	5.618446	-1.320963	2.478128
C	4.504357	-0.834699	3.159360
C	3.433927	-0.234406	2.478322
C	3.452394	-0.122140	1.066484
C	4.610507	-0.596299	0.378064
H	6.534685	-1.543557	0.538152
C	6.764883	-1.965877	3.214751
H	4.466677	-0.919100	4.246582
C	2.297263	0.343885	3.289846
C	4.706850	-0.466445	-1.127785
H	2.604449	1.282065	3.772786
H	1.439563	0.568823	2.643331
H	1.965780	-0.339348	4.082145
H	5.698355	-0.763105	-1.489292
H	3.963607	-1.099765	-1.636283
H	4.524171	0.571197	-1.441893
H	6.972685	-2.969231	2.823805
H	7.685201	-1.379097	3.103751
H	6.549255	-2.055328	4.284566
Cu	2.332540	1.277988	0.221352
C	2.302652	4.156284	-2.708510
C	2.454337	5.350788	-1.992497
C	2.227692	5.334316	-0.614494
C	1.874818	4.155543	0.053630
C	1.732137	2.939943	-0.666232
C	1.938649	2.968032	-2.072263
H	2.464415	4.159730	-3.787438
C	2.877648	6.617289	-2.692379
H	2.332110	6.260499	-0.047068
C	1.588912	4.218435	1.539276
C	1.710537	1.724120	-2.901659
H	2.254687	4.926700	2.047360
H	0.553256	4.542009	1.724388
H	1.711518	3.230716	2.007989
H	2.110629	1.836077	-3.916357
H	2.172574	0.837834	-2.442235
H	0.634627	1.512032	-2.987507
H	3.970651	6.670285	-2.779502
H	2.467181	6.668093	-3.707028
H	2.548509	7.505140	-2.141723
Cu	-0.000947	2.117048	0.000307
C	-2.233845	5.331419	0.615308
C	-2.460751	5.347315	1.993251
C	-2.307679	4.152830	2.709047
C	-1.942038	2.965199	2.072628
C	-1.735207	2.937685	0.666608
C	-1.879300	4.153231	-0.053010
H	-2.339391	6.257573	0.048048
C	-2.885714	6.613084	2.693458
H	-2.469679	4.155830	3.787944
C	-1.712521	1.721410	2.901815

C	-1.593317	4.216801	-1.538612
H	-2.113105	1.832608	3.916402
H	-2.173200	0.834605	2.442041
H	-0.636358	1.510766	2.987982
H	-2.260193	4.924061	-2.046657
H	-0.558181	4.542097	-1.723590
H	-1.714277	3.228951	-2.007482
H	-2.561693	7.501474	2.140656
H	-3.978542	6.662403	2.784802
H	-2.471526	6.666336	3.706478
Cu	-2.333381	1.275205	-0.221452
C	-4.503790	-0.839401	-3.159225
C	-5.617606	-1.326251	-2.477851
C	-5.659236	-1.188312	-1.081662
C	-4.609806	-0.601025	-0.377904
C	-3.452298	-0.125768	-1.066471
C	-3.433908	-0.238070	-2.478365
H	-4.465943	-0.924275	-4.246410
C	-6.765056	-1.968883	-3.214883
H	-6.532922	-1.550587	-0.537709
C	-4.705921	-0.471747	1.128005
C	-2.297751	0.341092	-3.289982
H	-5.697080	-0.769407	1.489636
H	-3.961994	-1.104535	1.636162
H	-4.524113	0.565968	1.442372
H	-1.965836	-0.341835	-4.082363
H	-2.605720	1.279057	-3.772838
H	-1.440150	0.566673	-2.643563
H	-7.674937	-1.360983	-3.134803
H	-6.535083	-2.091171	-4.278457
H	-6.999211	-2.956647	-2.800173
Cu	-1.958614	-1.153821	-0.144804
C	-3.659327	-4.211743	1.452272
C	-3.556693	-3.947537	2.823131
C	-2.518949	-3.123122	3.270890
C	-1.601771	-2.563363	2.375714
C	-1.703148	-2.824501	0.984421
C	-2.751819	-3.672024	0.536535
H	-4.467787	-4.850691	1.093600
C	-4.528586	-4.561675	3.798609
H	-2.431044	-2.908962	4.337029
C	-0.478817	-1.691545	2.891145
C	-2.920671	-3.957028	-0.941642
H	0.460105	-2.260472	2.983470
H	-0.283276	-0.870508	2.186925
H	-0.707145	-1.258232	3.873745
H	-3.527044	-4.855015	-1.110057
H	-3.418342	-3.118794	-1.454409
H	-1.943871	-4.102986	-1.423252
H	-5.521165	-4.680281	3.350196
H	-4.191232	-5.557630	4.113870

H	-4.627974	-3.948353	4.700777

Heptamer

Cu	2.917189	-0.148959	0.676689
C	2.465275	-3.116812	3.660964
C	3.464941	-4.036998	3.336570
C	4.136917	-3.892990	2.115230
C	3.816664	-2.867645	1.223531
C	2.789845	-1.934711	1.545158
C	2.132765	-2.070343	2.792267
H	1.934823	-3.217244	4.609039
C	3.833667	-5.147495	4.287375
H	4.919173	-4.606610	1.852265
C	4.560103	-2.757987	-0.091330
C	1.089373	-1.057217	3.196269
H	5.325070	-3.537837	-0.183123
H	3.875305	-2.855087	-0.948232
H	5.056511	-1.780211	-0.178475
H	1.563736	-0.158831	3.617777
H	0.510767	-0.735022	2.318570
H	0.393557	-1.450962	3.948201
H	4.720414	-4.881302	4.877129
H	3.020023	-5.355164	4.990575
H	4.067889	-6.072128	3.747911
Cu	1.471452	-2.033629	0.039694
C	2.187836	-5.156008	-2.163478
C	2.632931	-4.543979	-3.341060
C	2.230330	-3.230744	-3.605614
C	1.390737	-2.538655	-2.726791
C	0.936403	-3.154366	-1.534406
C	1.357490	-4.484815	-1.261372
H	2.508420	-6.174662	-1.940322
C	3.512157	-5.291287	-4.311411
H	2.581812	-2.738161	-4.513330
C	0.975822	-1.120041	-3.037465
C	0.939252	-5.168838	0.024307
H	1.526699	-0.712041	-3.894393
H	-0.099847	-1.057371	-3.260965
H	1.157332	-0.467210	-2.169801
H	1.295073	-6.205227	0.058133
H	1.349688	-4.644317	0.902224
H	-0.155443	-5.178760	0.131854
H	4.167454	-5.998810	-3.791463
H	2.908186	-5.868033	-5.023929
H	4.137159	-4.604270	-4.892279
Cu	-0.795199	-2.652003	-0.703222
C	-3.279024	-4.811329	1.612322
C	-4.086912	-5.439236	0.657455
C	-4.154950	-4.888281	-0.625503
C	-3.432814	-3.739614	-0.964194
C	-2.603906	-3.105784	-0.001356

C	-2.541061	-3.666171	1.301659
H	-3.223049	-5.228699	2.618792
C	-4.846603	-6.696064	0.999285
H	-4.788859	-5.363385	-1.375654
C	-3.572570	-3.153447	-2.352599
C	-1.644486	-3.047442	2.350303
H	-3.987083	-3.882647	-3.058474
H	-4.238913	-2.277681	-2.341144
H	-2.598782	-2.815791	-2.738298
H	-1.867138	-3.433220	3.352584
H	-0.583644	-3.259757	2.138172
H	-1.751288	-1.953217	2.365603
H	-4.227090	-7.587081	0.833688
H	-5.152632	-6.702045	2.051257
H	-5.743237	-6.800217	0.378715
Cu	-2.689687	-1.115983	-0.164779
C	-6.373383	0.444924	0.107750
C	-6.704655	0.611090	-1.243257
C	-5.671140	0.666075	-2.180661
C	-4.329006	0.557300	-1.795821
C	-3.991105	0.375774	-0.432384
C	-5.045960	0.326671	0.520979
H	-7.171828	0.400658	0.849976
C	-8.147575	0.706255	-1.670503
H	-5.916741	0.796446	-3.235597
C	-3.247710	0.654837	-2.848291
C	-4.728409	0.155144	1.990993
H	-3.633818	0.418823	-3.847204
H	-2.824795	1.669003	-2.883764
H	-2.416220	-0.030245	-2.623769
H	-5.637797	0.004290	2.584070
H	-4.069256	-0.711883	2.151300
H	-4.207914	1.040386	2.387353
H	-8.649978	-0.265386	-1.582010
H	-8.701347	1.415156	-1.043945
H	-8.232378	1.032054	-2.712580
Cu	-2.407719	1.345957	0.283427
C	-2.436655	3.636699	3.606245
C	-3.146501	4.753860	3.148542
C	-3.202869	4.988120	1.772892
C	-2.582457	4.128999	0.858024
C	-1.877666	2.984939	1.317792
C	-1.807952	2.762792	2.717539
H	-2.374112	3.448113	4.678934
C	-3.851414	5.666923	4.119511
H	-3.743476	5.860900	1.403722
C	-2.638132	4.460490	-0.618000
C	-1.045478	1.571513	3.244990
H	-3.491408	5.108346	-0.851006
H	-1.721954	4.977696	-0.940628
H	-2.723326	3.546839	-1.225714

H	-1.046752	1.535049	4.340839
H	-1.476167	0.629701	2.869500
H	-0.002468	1.600272	2.899617
H	-4.841510	5.272251	4.382138
H	-3.284492	5.770620	5.051469
H	-3.997940	6.664756	3.692232
Cu	-0.287823	2.640130	0.164885
C	1.189589	5.974649	-1.282423
C	0.716230	5.934432	-2.599146
C	0.384345	4.692698	-3.146025
C	0.522737	3.509466	-2.411536
C	1.004715	3.541760	-1.078783
C	1.340499	4.810025	-0.525927
H	1.439174	6.938319	-0.836057
C	0.582960	7.199294	-3.408511
H	-0.002810	4.646902	-4.164997
C	0.151505	2.197659	-3.060044
C	1.867902	4.910124	0.888784
H	-0.559528	2.338999	-3.883328
H	1.039093	1.696958	-3.471052
H	-0.295401	1.511878	-2.325684
H	1.915262	5.952033	1.226047
H	1.227550	4.353834	1.590741
H	2.879169	4.483375	0.959488
H	0.282290	8.044657	-2.779561
H	1.538075	7.466391	-3.879053
H	-0.156545	7.083762	-4.208254
Cu	2.094382	2.045495	-0.331423
C	5.806205	2.598541	1.233499
C	6.597745	2.472585	0.089941
C	6.063431	1.816843	-1.027941
C	4.762048	1.314172	-1.021454
C	3.948135	1.452265	0.136188
C	4.501659	2.091255	1.274295
H	6.218174	3.093864	2.114074
C	7.992085	3.044610	0.045613
H	6.678636	1.704833	-1.922011
C	4.206591	0.634402	-2.254799
C	3.709607	2.174958	2.562616
H	4.964203	0.546428	-3.042258
H	3.831656	-0.374010	-2.022012
H	3.357573	1.203410	-2.664083
H	4.000047	3.044344	3.165345
H	2.630444	2.238862	2.357058
H	3.870937	1.275738	3.175849
H	8.006899	4.002592	-0.489990
H	8.381083	3.222939	1.053733
H	8.681742	2.370721	-0.475253
